# YiQi-HuoXue prescription ameliorates LPS-induced sepsis-associated encephalopathy via VCAM-1–mediated microglial efferocytosis

**DOI:** 10.3389/fimmu.2026.1792688

**Published:** 2026-04-01

**Authors:** Ziming Wang, Enfeng Song, Yang Wang, Yun Lu

**Affiliations:** 1 School of Clinical Medicine, Chengdu University of TCM, Chengdu, Sichuan, China; 2 Emergency Department, Hospital of Chengdu University of TCM, Chengdu, Sichuan, China; 3 Department of TCM, Renmin Hospital of Wuhan University, Wuhan, Hubei, China; 4 Department of Emergency Medicine Center, Sichuan Provincial People’s Hospital, Chengdu, Sichuan, China; 5 University of Electronic Science and Technology of China, Chengdu, Sichuan, China

**Keywords:** efferocytosis, sepsis-associated encephalopathy (SAE), traditional Chinese medicine, VCAM-1, YiQi-HuoXue prescription

## Abstract

**Background:**

The YiQi-HuoXue therapeutic approach, derived from traditional Chinese medicine, has long been used to invigorate Qi, promote blood circulation, and restore brain function in various neurological and cerebrovascular disorders. Despite its extensive clinical application, the therapeutic potential and underlying mechanisms of YiQi-HuoXue in sepsis-associated encephalopathy (SAE)—a neuroinflammatory condition with no effective treatment—remain insufficiently elucidated.

**Objective:**

To test whether YiQi-HuoXue Prescription (YQHXP) mitigates SAE and to define a mechanism centered on endothelial VCAM-1 and microglial efferocytosis.

**Methods:**

LPS-challenged mice received oral YQHXP and were assessed by Morris water maze (MWM) and open-field tests (OFT). Hippocampal tumor necrosis factor alpha (TNF-α), interleukin-6 (IL-6), and microglial activation were quantified by enzyme-linked immunosorbent assay (ELISA) and immunostaining. YQHXP constituents and plasma-exposed prototype constituents were profiled by UPLC-HRMS. Network pharmacology integrated with hippocampal RNA-seq nominated VCAM-1–efferocytosis nodes. *In vitro*, efferocytosis (PKH67^+^/F4/80^+^), apoptosis, and cytokine release were measured, with pathway relevance probed using a MER proto-oncogene tyrosine kinase (MERTK) inhibitor (UNC2250) and a VCAM-1 suppressor (rutin). VCAM-1–ligand interactions were evaluated by docking and 100-ns molecular dynamics (MD) simulations, and binding was confirmed by surface plasmon resonance (SPR).

**Results:**

YQHXP improved spatial learning and novelty-directed exploration, and reduced hippocampal TNF-α, IL-6, and microglial activation. Analyses converged on a VCAM-1–linked efferocytosis axis involving complement C1q subcomponent B (C1QB)/MERTK. YQHXP downregulated VCAM-1, upregulated C1QB and MERTK in hippocampus and BV2 cells, enhanced efferocytosis, and decreased microglial apoptosis and cytokine release. Rutin or YQHXP alone increased efferocytosis; YQHXP partially restored clearance under MERTK inhibition, consistent with VCAM-1–gated regulation. Docking/MD predicted VCAM-1 engagement by several plasma-exposed prototypes, and SPR confirmed sub-millimolar binding between VCAM-1 and ginsenoside Rg1.

**Discussion:**

YQHXP may attenuate experimental SAE via VCAM-1–mediated enhancement of microglial efferocytosis, supporting VCAM-1 as a pharmacodynamic marker and efferocytosis as an actionable therapeutic axis.

## Graphical Abstract

**Figure:**
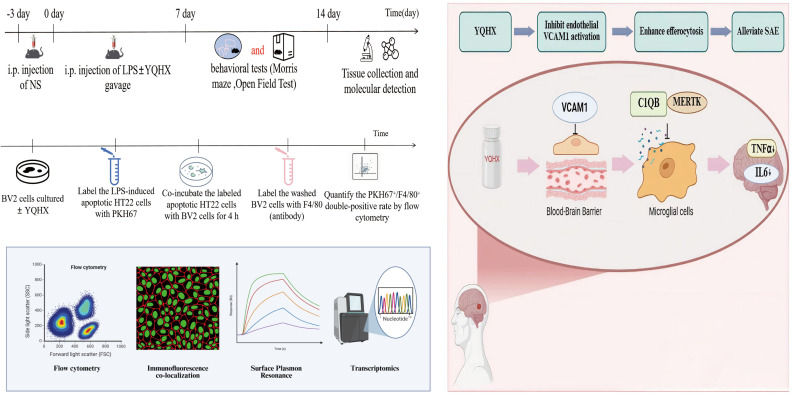

## Introduction

Sepsis-associated encephalopathy (SAE) is an acute, diffuse brain dysfunction secondary to systemic infection that occurs without overt central nervous system infection (1, 2). Clinically, SAE manifests as altered consciousness, cognitive impairment, seizures, or coma, with delirium commonly observed (3). Prevalence among ICU sepsis cohorts ranges from 9–76% (4), and incidence in other series is estimated at ~20–40% (5). Management remains largely supportive—prompt antimicrobials, fluid resuscitation, organ support, and judicious sedation—and no disease-modifying therapy has been established (6, 7). Moreover, current approaches have important limitations: aggressive fluids can worsen metabolic and renal dysfunction; antibiotics in children carry risks and do not directly address neuroinflammation; and sedatives complicate neurological assessment and introduce systemic adverse effects (8–11). These gaps underscore an urgent need for mechanism-informed therapeutic strategies.

The YiQi-HuoXue Prescription is a TCM formula that originates from the Qing-dynasty physician Wang Qingren’s doctrine of “invigorating Qi and activating blood”. It comprises Ginseng Radix et Rhizoma Rubra (red ginseng; steamed root and rhizome of *Panax ginseng* C.A. Mey.), Notoginseng Radix et Rhizoma (*Panax notoginseng* (Burkill) F.H. Chen), Chuanxiong Rhizoma (*Ligusticum chuanxiong* Hort.), and Rhei Radix et Rhizoma (commonly sourced from *Rheum palmatum* L.). Grounded in TCM theory, the formula is intended to replenish Qi, promote blood circulation, and detoxify pathogenic factors and, over the past decades, has been used predominantly for the treatment of stroke and cognitive impairment. Nevertheless, its molecular basis in inflammation-driven neurological disease, particularly SAE, remains insufficiently defined. Emerging mechanistic studies show that YQHXP suppresses NF-κB/NLRP3 inflammasome activation, downregulates p-NF-κB p65, NLRP3 and other pyroptosis-related proteins, reduces circulating IL-1β, IL-18 and IL-6, and ameliorates neurological deficits in experimental models (12, 13). Building on its anti-inflammatory and neuroprotective profile that is most apparent in inflammation-dominant settings, the present study focuses on SAE—whose core pathophysiology is inflammatory—to systematically evaluate the therapeutic efficacy of YQHXP and elucidate its mechanisms, employing models that more faithfully recapitulate inflammation-centric neuropathology to assess translational potential.

Efferocytosis, the programmed recognition and removal of apoptotic cells by professional phagocytes, acts as a critical brake on neuroinflammation and a prerequisite for tissue repair in the brain. In the central nervous system, microglia coordinate this clearance to prevent secondary necrosis and to restore network homeostasis (14, 15). When efferocytosis fails, inflammatory cascades persist and synaptic injury worsens (16, 17). In SAE, hippocampal efferocytic capacity is diminished, and strengthening the clearance pathway mediated by opsonins and integrins restores microglial removal of apoptotic cells, lowers TNF-α and IL-6, and improves behavioral outcomes (18). More broadly, current models of SAE emphasize systemic cytokinemia, disruption of the blood-brain barrier (BBB), microglial activation, and maladaptive innate-immune signaling. These conditions set the stage for defective efferocytosis to aggravate neuronal injury and cognitive decline (7, 19).

Among regulators at the neurovascular interface that intersect with microglial clearance, vascular cell adhesion molecule-1 (VCAM-1) has drawn particular attention. VCAM-1 is a cytokine-inducible endothelial receptor that marks vascular activation and orchestrates leukocyte adhesion. In sepsis, circulating VCAM-1 rises and associates with adverse outcomes; admission VCAM-1 independently predicts septic encephalopathy in community-onset severe sepsis (20). In the brain, endothelial VCAM-1 accompanies BBB dysfunction and promotes immune-cell trafficking, and VCAM-1 liganding has been used to target inflamed cerebral vasculature (21, 22). Taken together, these data implicate VCAM-1 as a mechanistic node in SAE and highlight it as a plausible therapeutic and biomarker target in inflammation-driven brain dysfunction (23).

Our group has conducted sustained clinical and mechanistic studies of YQHXP over many years (12, 24), establishing a solid foundation for translation. Prior work indicates anti-inflammatory and neuroprotective actions in preclinical settings (12, 13), yet relevance to SAE has remained undefined. Here, we provide a systematic assessment of YQHXP in SAE by profiling formulation constituents and plasma-exposed prototypes with UPLC–HRMS and by integrating network pharmacology with hippocampal transcriptomics to converge on intervention-ready targets. We then validate these targets in complementary *in vivo* and *in vitro* SAE models and use targeted perturbations to test causal relevance. Together, these findings establish a mechanistic basis for YQHXP as a translational candidate for the prevention and treatment of SAE.

## 2 Materials and methods

### 2.1 Materials

YQHXP (YiQi-HuoXue Prescription; batch no. 20240508) was procured from the Hospital of Chengdu University of Traditional Chinese Medicine (Chengdu, China). Mouse TNF-α (Cat. E-EL-M3063) and IL-6 (Cat. E-EL-M0044) ELISA kits were sourced from Elabscience Biotechnology Co., Ltd. (Wuhan, China). The Cell Counting Kit-8 (CCK-8; Cat. C0042) and FITC–Annexin V Apoptosis Detection Kit (Cat. C1062L) were obtained from Beyotime Biotechnology (Shanghai, China). Primary antibodies included anti-MERTK rabbit polyclonal (Cat. 27900-1-AP; Proteintech, Wuhan, China), anti-C1QB rabbit polyclonal (Cat No. 16919-1-AP; Proteintech Biotechnology, Wuhan, China), anti-IL6 rabbit polyclonal (Cat. GB11117; Servicebio,Wuhan, China), anti-TNFαrabbit polyclonal (Cat. GB115702; Servicebio,Wuhan, China), anti-lBA1 rabbit polyclonal (Cat. GB15105; Servicebio,Wuhan, China)and anti-VCAM-1 rabbit polyclonal (Cat. 381014; Zenbio, Chengdu, China). The MERTK inhibitor UNC2250 (Cat. HY-15797), rutin (Cat. HY-N0148), and Ginsenoside Rg1 (Cat. HY-N0045) were purchased from MedChemExpress (Monmouth Junction, NJ, USA). PKH67 dye (Cat. MINI67) and lipopolysaccharide (LPS; Escherichia coli O55:B5; Cat. L2880) were from Sigma-Aldrich (St. Louis, MO, USA). The F4/80 antibody (Cat. 123116) was from BioLegend (San Diego, CA, USA). Recombinant human VCAM-1 protein (Cat. VC1-H5224) was obtained from ACROBiosystems (Newark, DE, USA). A CM5 sensor chip (Cat. 29104988) was supplied by Cytiva (Uppsala, Sweden).

### 2.2 Animals and model establishment

Male C57BL/6 mice (8 weeks; n = 25) and male Sprague–Dawley rats (8 weeks; n = 40) were obtained from the Experimental Animal Center of Sichuan Provincial People’s Hospital (Chengdu, China). All procedures were approved by the Ethics Committee of Sichuan Provincial People’s Hospital (No. 661, 2024) and complied with institutional and national guidelines for the care and use of laboratory animals. Animals were housed under SPF conditions with free access to food and water. Before the experiment, mice and rats were singly housed for 1 week to acclimate to the facility.

For the *in vivo* SAE model, mice were randomly assigned to Control, Model (LPS), or YQHXP treatment groups (Low/Medium/High). Except for the Control group, mice received an intraperitoneal injection of LPS (10 mg·kg^-1^) (25) to induce sepsis; Control mice received an equal volume of sterile saline.To document model induction and implement quality control prior to intervention, we performed longitudinal clinical monitoring within the first 24 h after LPS challenge. Disease severity was quantified using the Murine Sepsis Score (MSS) as previously described (26), and rectal temperature and body weight were measured in parallel. Specifically, MSS, rectal temperature, and body weight were recorded at 2, 4, 8, 12, and 24 h after LPS (or saline) injection in the Model and Control groups. The time-course data for MSS, temperature, and body weight during the pre-intervention window (0–24 h) are presented as line plots in the **Supplementary Materials** (**Supplementary Figure S1**).

YQHXP was administered by oral gavage once daily at 6.5, 13, or 26 g·kg^-1^ (Low/Medium/High, respectively) (27) for 7 consecutive days. To ensure that treatment initiation occurred after model stabilization and baseline documentation, the first dose was given 24 h after LPS injection, and subsequent doses were administered at the same time each day. Control and Model groups received vehicle (saline) by gavage, with the same volume as the YQHXP Medium-dose group. All clinical scoring and downstream assessments were performed by investigators blinded to group allocation.

Rats were used exclusively to prepare YQHXP–containing serum for *in vitro* experiments; the preparation and qualification of drug-containing serum are described in the corresponding Methods subsection.

### 2.3 Cell culture

BV2 microglia (Cat. CL-0493) and HT22 hippocampal neurons (Cat. CL-0697) were obtained from Procell Life Science & Technology Co., Ltd. (Wuhan, China). Cells were cultured in Dulbecco’s modified Eagle’s medium (DMEM) supplemented with 10% fetal bovine serum (FBS) and penicillin–streptomycin (100 U/mL penicillin, 100 μg/mL streptomycin) at 37 °C in a humidified atmosphere with 5% CO_2_.

### 2.4 Preparation of YQHXP–containing serum

Forty male Sprague–Dawley rats (8 weeks) were randomly allocated to four groups: Control, YQHXP–High, YQHXP–Medium, and YQHXP–Low. Rats were used exclusively for drug-containing serum preparation to obtain sufficient serum volume with fewer animals, consistent with the 3Rs principle. Rats in the YQHXP groups received YQHXP by oral gavage once daily for 7 consecutive days at 18, 9, or 4.5 mL·kg^-1^·day^-1^ (High/Medium/Low, respectively) (28). The Control group received physiological saline by gavage at a volume equivalent to the Medium dose (9 mL·kg^-1^·day^-1^).

On day 7, 1 h after the final gavage, rats were anesthetized with isoflurane and blood was collected aseptically from the abdominal aorta. Whole blood was allowed to clot at room temperature for 30–40 min and then centrifuged at 3,000 × g for 10 min at 4 °C. Serum was carefully harvested, and visibly hemolyzed samples were excluded. Serum was pooled within each group, heat-inactivated at 56 °C for 30 min, and filtered through a sterile 0.22-μm membrane. Aliquots were stored at −80 °C and subjected to a single freeze–thaw cycle at most before use.

To minimize potential bias unrelated to YQHXP exposure, Control serum and YQHXP-containing serum were processed in parallel using identical procedures (collection, clotting, centrifugation, heat inactivation, filtration, storage, and thawing). In subsequent *in vitro* experiments, drug-containing serum was compared against the matched Control serum prepared from vehicle-treated rats, so that the primary systematic difference between sera reflected exposure to YQHXP rather than sample handling.

### 2.5 Tumor necrosis factor alpha and interleukin-6 quantification

TNF-α and IL-6 were quantified in mouse hippocampal extracts and in cell-culture supernatants using commercial enzyme-linked immunosorbent assay (ELISA) kits in accordance with the manufacturer’s instructions. Briefly, hippocampi were rinsed in ice-cold PBS (pH 7.4), minced, and homogenized on ice in PBS at ~1:9 (w/v); homogenates were clarified (5,000 × g, 5–10 min, 2–8 °C) and the supernatants collected. Conditioned media were cleared by centrifugation (1,000 × g, 20 min, 2–8 °C). Standards were prepared by serial dilution; 100 µL of standards, blanks, or diluted samples were added to pre-coated wells and incubated at 37 °C for 90 min. After decanting, 100 µL biotinylated detection antibody was added (37 °C, 1 h), plates were washed 3×, 100 µL HRP conjugate was added (37 °C, 30 min), followed by 5× washes. TMB substrate was then applied (37 °C, protected from light, ~15 min), the reaction was stopped with 50 µL stop solution, and absorbance was read at 450 nm. Concentrations were calculated from standard curves fitted with a four-parameter logistic model and reported as pg/mg tissue (tissue extracts) or pg/mL (supernatants). Samples outside the linear range were re-assayed after further dilution; repeated freeze–thaw cycles were avoided.

### 2.6 Morris water maze

The MWM was conducted over seven consecutive days with salient extra-maze cues. Day 1 used a visible platform (acclimation/health check). Days 2–6 consisted of hidden-platform training with the platform kept at a fixed location; mice performed multiple trials per day from semi-random start points. On Day 7, a probe trial was run with the platform removed. Water was rendered opaque and maintained at a constant temperature, trajectories were captured by video-tracking, and animals were gently dried between trials by personnel blinded to group allocation. From the tracking outputs, the escape latency on the final training day and the time spent in the target quadrant during the probe were extracted for downstream analyses (29).

### 2.7 Open-field test

The OFT was conducted in a walled square arena under uniform illumination with salient visual cues. Each mouse was placed gently into the arena and allowed to explore for a fixed interval while locomotion was recorded by a video-tracking system. A center zone (region of interest) was predefined in the analysis software, and the time spent in the center zone expressed as a percentage of total session time (% time in center) was calculated. The arena was cleaned and dried between trials to remove olfactory residues. All recordings and quantifications were performed with investigators blinded to group allocation.

### 2.8 Immunofluorescence for hippocampal Iba1 and colocalization with TNF-α/IL-6

Formalin-fixed, paraffin-embedded hippocampal sections were processed for immunofluorescence. After dewaxing to water through xylene-free clearing reagent and graded ethanols, heat-induced epitope retrieval was performed in EDTA buffer (pH 8.0) using a microwave program. Sections were washed in PBS and blocked for 30 min. For double labeling, mouse Iba1 (1:200) was combined with rabbit IL-6 (1:100) or rabbit TNF-α (1:200), applied overnight at 4 °C. After PBS washes, secondary antibodies were added for 50 min at room temperature: CY3-conjugated goat anti-mouse IgG (1:300) together with Alexa Fluor 488-conjugated goat anti-rabbit IgG (1:400). Nuclei were counterstained with DAPI (10 min); tissue autofluorescence was quenched and slides were mounted with antifade medium. Images were acquired on a Nikon Eclipse C1 fluorescence microscope and/or scanned on a 3DHISTECH Pannoramic MIDI system.

### 2.9 Immunohistochemistry for hippocampal Complement C1q Subcomponent B, MER proto-oncogene, tyrosine kinase, and VCAM-1

Formalin-fixed, paraffin-embedded hippocampal tissues were sectioned and processed for chromogenic IHC. Sections were deparaffinized to water with xylene-free clearing reagent and graded ethanols, followed by heat-induced epitope retrieval in citrate buffer, pH 6.0 using a microwave program. Endogenous peroxidase was quenched with 3% H_2_O_2_ (25 min, RT), and nonspecific binding blocked with 3% BSA. Primary antibodies were applied overnight at 4 °C: anti-C1QB (1:200), anti-MERTK (1:200), and anti-VCAM-1 (1:100). After PBS washes, sections were incubated with HRP-conjugated goat anti-rabbit IgG (≈50 min, RT), developed with DAB chromogen, counterstained with hematoxylin, differentiated, blued, dehydrated through graded alcohols/clearing agents, and coverslipped. Bright-field images were acquired on a Nikon microscope under identical settings across groups.

### 2.10 Composition profiling of YQHXP and identification of plasma-absorbed constituents

#### 2.10.1 Composition profiling of YQHXP (UPLC–Q-TOF-MS)

YQHXP was profiled by UPLC–Q-TOF-MS. Briefly, an aliquot of the preparation (1 mL) was diluted with 20 mL of 20% methanol, vortexed, and centrifuged (12,000 rpm, 5 min). The supernatant was analyzed on a Waters ACQUITY UPLC HSS T3 column (2.1 × 100 mm, 1.8 μm) at 30 °C and 0.3 mL·min^-1^ using mobile phases A (acetonitrile) and B (0.1% formic acid in water) under a standard gradient. MS data were acquired on an AB Sciex TripleTOF 4600 in ESI positive/negative modes (typical settings: TOF m/z 50–1700, source temperature 500 °C, curtain and nebulizing gases per instrument recommendations). Data were processed with Analyst TF and PeakView; tentative identifications were assigned by matching high-resolution MS/MS spectra to a curated natural-products library and literature, followed by manual inspection of diagnostic fragments.

#### 2.10.2 Plasma-absorbed constituents after oral administration

To characterize blood-borne exposures, blank serum and dosed serum were prepared in parallel. Serum proteins were precipitated by adding 3× volume of methanol (MS grade), vortexing (≈5 min), standing at 4 °C (15 min), and centrifuging (12,000 rpm, 15 min). Supernatants were dried, stored at −80 °C, and reconstituted in 50% methanol (100 μL) prior to analysis; an injection volume of 5 μL was used. Chromatographic and MS conditions matched those above.

### 2.11 Transcriptome sequencing and data analysis

Total RNA was extracted from hippocampal tissue using the TRIzol method (chloroform phase separation, isopropanol precipitation, 75% ethanol wash), and resuspended in RNase-free water. Quantity and purity were assessed by NanoDrop 2000, and integrity by Agilent 2100 Bioanalyzer; only samples with RIN ≥ 7 proceeded to library construction. Poly(A)^+^ mRNA was enriched with oligo(dT) magnetic beads and fragmented under divalent-cation conditions, followed by first- and second-strand cDNA synthesis (NEBNext Ultra Directional RNA Library Prep Kit for Illumina). After purification, end repair, 3′ A-tailing, and adapter ligation, fragments of approximately 400–500 bp were selected with AMPure XP, PCR-amplified, purified, and quality-checked (Agilent 2100 High Sensitivity DNA). Equimolar libraries were pooled and sequenced on an Illumina platform in paired-end 150 bp (PE150) mode.

Raw FASTQ files were filtered with fastp v0.22.0 (adapter trimming; removal of low-quality reads, mean Q < 20) and aligned to the reference mouse genome using HISAT2 v2.1.0. Gene-level counts were generated with HTSeq v0.9.1 and normalized as FPKM. Differentially expressed genes (DEGs) were identified by DESeq v1.38.3. Functional interpretation included GO enrichment (topGO v2.50.0, hypergeometric test, P < 0.05), KEGG pathway analysis (clusterProfiler v4.6.0, P < 0.05), and GSEA v4.1.0 on ranked gene lists. Where indicated, extended analyses followed common practice: transcript assembly (StringTie v2.2.1), alternative splicing (rMATS v4.0.1; SE/RI/MXE/A5SS/A3SS), variant calling (VarScan v2.3.9 with stringent quality/coverage filters), exon-usage testing (DEXSeq v1.44.0), transcription-factor annotation (AnimalTFDB), and PPI network construction (STRING, high-confidence edges).

### 2.12 Network pharmacology

Plasma-absorbed prototypes identified in Section 2.10 were used as the chemical input. For each compound, canonical structures (SMILES/InChIKey) were confirmed against public repositories and harmonized to avoid duplicates. Putative protein targets were retrieved from SwissTargetPrediction (organism = Homo sapiens), and the union of predicted targets per compound was consolidated to HGNC gene symbols via UniProt mapping. Disease-related genes for SAE were compiled from GeneCards and OMIM. After de-duplication and ID harmonization, compound targets and disease genes were intersected to obtain common targets (CTs); the intersection was visualized with a Venn diagram.Protein–protein interactions (PPIs) among CTs were queried in STRING (Homo sapiens; high-confidence setting) and exported to Cytoscape for network visualization. Functional annotation of CTs used clusterProfiler (R): KEGG pathway and GO (BP/CC/MF) enrichment analyses were performed with Benjamini–Hochberg multiple-testing correction, and terms with adjusted P values < 0.05 were retained.

### 2.13 Flow cytometry for apoptosis and efferocytosis

BV2 cells were harvested after treatments, washed twice with cold PBS, and resuspended in 1× binding buffer (from the FITC–Annexin V kit),. Cells were stained with Annexin V–FITC and propidium iodide (PI) for 10–15 min at room temperature in the dark and analyzed within 1 h on a benchtop flow cytometer. A standard gating strategy was applied: FSC/SSC to exclude debris, singlet gating, then quantification of early apoptosis (Annexin V^+^/PI^–^) and late apoptosis/necrosis (Annexin V^+^/PI^+^); the apoptotic fraction was defined as Annexin V^+^ (early ^+^ late). Unstained, single-stained, and fluorescence-minus-one controls were used for compensation and thresholding; analysis was performed in FlowJo.

HT22 neurons were rendered apoptotic (per experimental design) and labeled with PKH67 following the manufacturer’s protocol; excess dye was quenched and removed by thorough washing. BV2 cells were co-cultured with PKH67^+^ apoptotic HT22 in a humidified 37 °C, 5% CO_2_ incubator for 4 h at a predefined BV2:HT22 ratio. Non-engulfed HT22 were removed by gentle washing, and BV2 were detached using enzyme-free dissociation buffer. Cell suspensions were blocked (optional) and stained with F4/80 for 20–30 min at 4 °C in the dark, washed, and acquired by flow cytometry. After FSC/SSC and singlet gating, BV2 were identified as F4/80^+^ events, and the efferocytosis rate was calculated as the PKH67^+^/F4/80^+^ double-positive percentage. Compensation was set using single-color controls (PKH67-only HT22; F4/80-only BV2) and fluorescence-minus-one controls; data were analyzed in FlowJo by operators blinded to group allocation (30).

### 2.14 Western blot

Hippocampal tissue and BV2 cells were lysed in ice-cold RIPA buffer supplemented with protease/phosphatase inhibitors and clarified by centrifugation (12,000 × g, 15 min, 4 °C). Protein concentration was determined by BCA assay. Equal amounts of protein (20–40 μg per lane) were denatured in Loading buffer at 95 °C for 5 min, separated by SDS–PAGE, and transferred onto PVDF membranes. Membranes were blocked in 5% non-fat milk/TBST for 1 h at room temperature, incubated overnight at 4 °C with primary antibodies. After washing, membranes were incubated with species-appropriate HRP-conjugated secondary antibodies (1:5000, 1 h, room temperature), developed with ECL substrate, and imaged on a chemiluminescence system. Band intensities were quantified in ImageJ, background-subtracted, normalized to the loading control, and expressed relative to the Control group; all analyses were performed blinded to group allocation.

### 2.15 Molecular docking and molecular dynamics simulations

Ligand 2D structures (Ginsenoside Rg1, Zingibroside R1, Ferulic acid, Rhein) were retrieved from PubChem and converted/minimized to 3D in ChemOffice 20.0 (.mol2). A high-resolution human VCAM-1 structure was downloaded from RCSB PDB and prepared in PyMOL 2.6.0 (removal of waters/ions, addition of hydrogens). Docking was performed in MOE 2019 using QuickPrep/SiteFinder, MMFF94x ligand minimization, Triangle-Matcher placement with London dG scoring and GBVI/WSA dG rescoring; 50 runs per ligand. Top poses were visualized in PyMOL/Discovery Studio 2019.

The best ligand–VCAM-1 complexes were simulated with GROMACS 2020.3 (periodic box, ≥1.0 nm padding, TIP3P water, 0.15 M NaCl). After energy minimization, NVT then NPT equilibration were applied (V-rescale 300 K, Parrinello–Rahman 1 bar). Production ran 100 ns (2 fs step), with PME electrostatics and 1.4 nm Lennard-Jones cutoff; bonds to H were constrained by LINCS. Analyses included RMSD, RMSF, radius of gyration, H-bond counts, and free-energy landscapes.

### 2.16 Surface plasmon resonance assay

SPR was performed on a Biacore T200 to assess binding between Ginsenoside Rg1 and human VCAM-1. Recombinant VCAM-1 (purity ≥90%) was immobilized on a CM5 chip via EDC/NHS amine coupling (protein at 25 µg/mL in sodium acetate, pH 4.5/5.0/5.5 optimized empirically) to ~10,000 RU, followed by 1 M ethanolamine blocking; a reference flow cell underwent activation/blocking without protein. Rhein, prepared in running buffer, was injected over the surface at 7.8125–500 µM (30 µL/min, 25 °C) with 60 s association and 60 s dissociation. Sensorgrams were processed by reference flow-cell subtraction with DMSO solvent correction, and kinetics were fit in Biacore T200 Evaluation Software v3.2 to obtain K_D.

### 2.17 Statistical analysis

Statistical analyses were performed in GraphPad Prism 8.0.1 (GraphPad Software) and SPSS 19.0 (IBM, IL, United States). Data are presented as mean ± SD with individual data points where appropriate. For comparisons among multiple groups, one-way ANOVA followed by Tukey’s multiple-comparisons test was used. Two-group comparisons used unpaired two-tailed Student’s t-tests. All tests were two-sided with P < 0.05 considered statistically significant.

## 3 Results

### YQHXP alleviated cognitive deficits in mice with SAE

**Figure 1:**
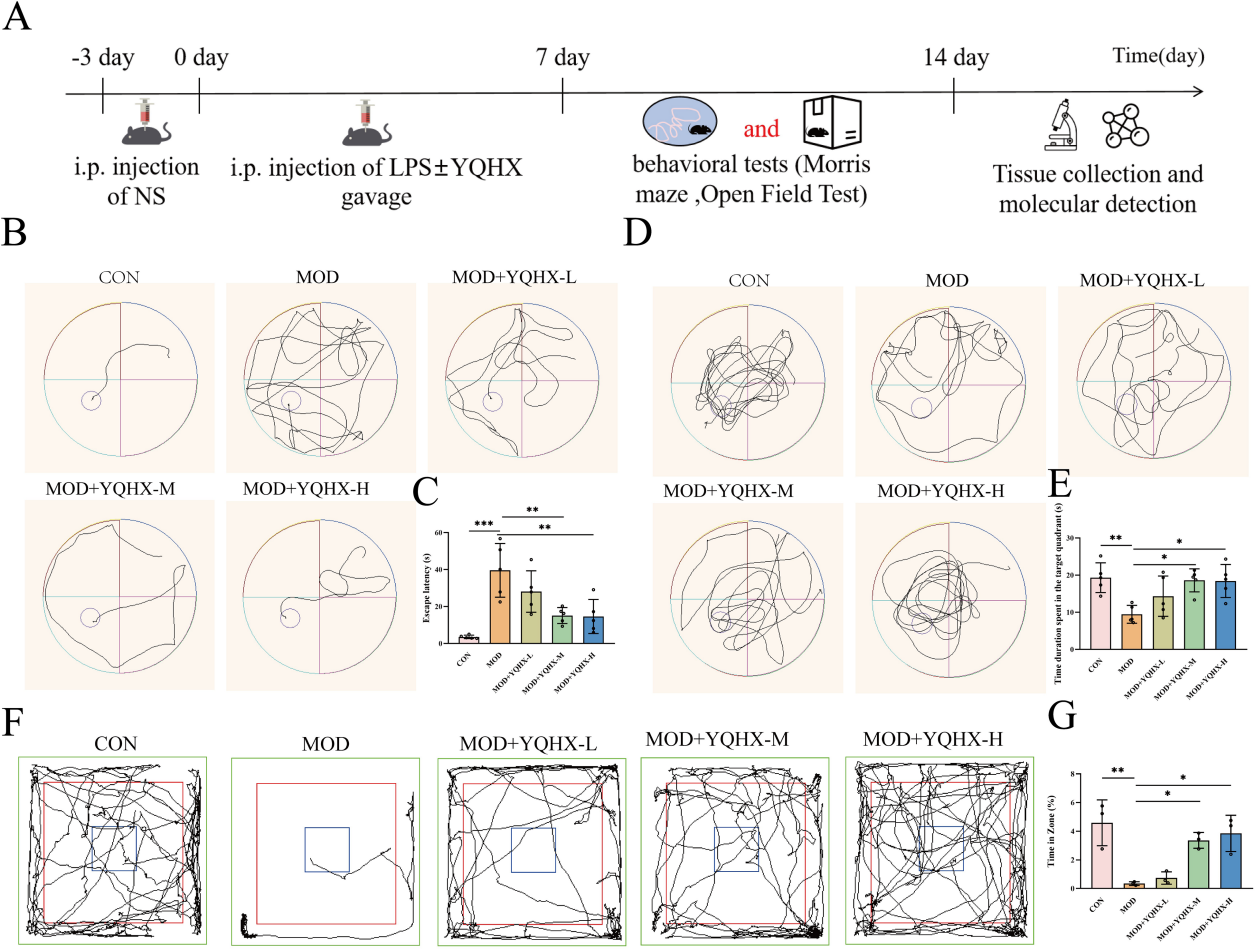
YQHXP reduces cognitive impairment in mice with SAE. **(A)** Study design and treatment timeline. **(B–C)** MWM acquisition (training phase). **(B)** Representative swim paths for each group. **(C)** Statistical plot summarizing escape latency; the Model group shows prolonged latency versus Control, whereas YQHXP medium and high doses shorten latency versus Model(n=5). **(D–E)** MWM probe test. **(D)** Representative search trajectories. **(E)** Statistical plot of time spent in the target quadrant; the Model group shows reduced target-quadrant time versus Control, while YQHXP medium and high doses increase target-quadrant time versus Model(n=5). **(F–G)** Open-field assay. **(F)** Representative exploration tracks. **(G)** Statistical plot of center-zone engagement; the Model group displays reduced exploration, whereas YQHXP medium and high doses significantly increase exploration versus Model(n=3). *P < 0.05 relative to the model group; **P < 0.01, ***P < 0.001 relative to the model group.

We adhered to the prespecified study design and dosing schedule (**Figure 1A**). During MWM training, model mice showed longer escape latencies than controls across days, indicating impaired spatial learning; medium and high doses of YQHXP significantly reduced latency relative to the model group (**Figures 1B, C**). In the probe test, model mice spent less time in the target quadrant, whereas YQHXP at medium and high doses increased target-quadrant time, indicating improved spatial memory (**Figures 1D, E**). In the OFT, model mice exhibited reduced exploratory behavior in a novel environment, reflected by decreased center-zone time; YQHXP (medium/high) significantly increased exploration versus the model group (**Figures 1F, G**). Together, these findings indicate that YQHXP alleviates SAE-related deficits in spatial learning and memory and enhances exploration.

### 3.2 YQHXP mitigates LPS-induced inflammation and neuronal injury

**Figure 2:**
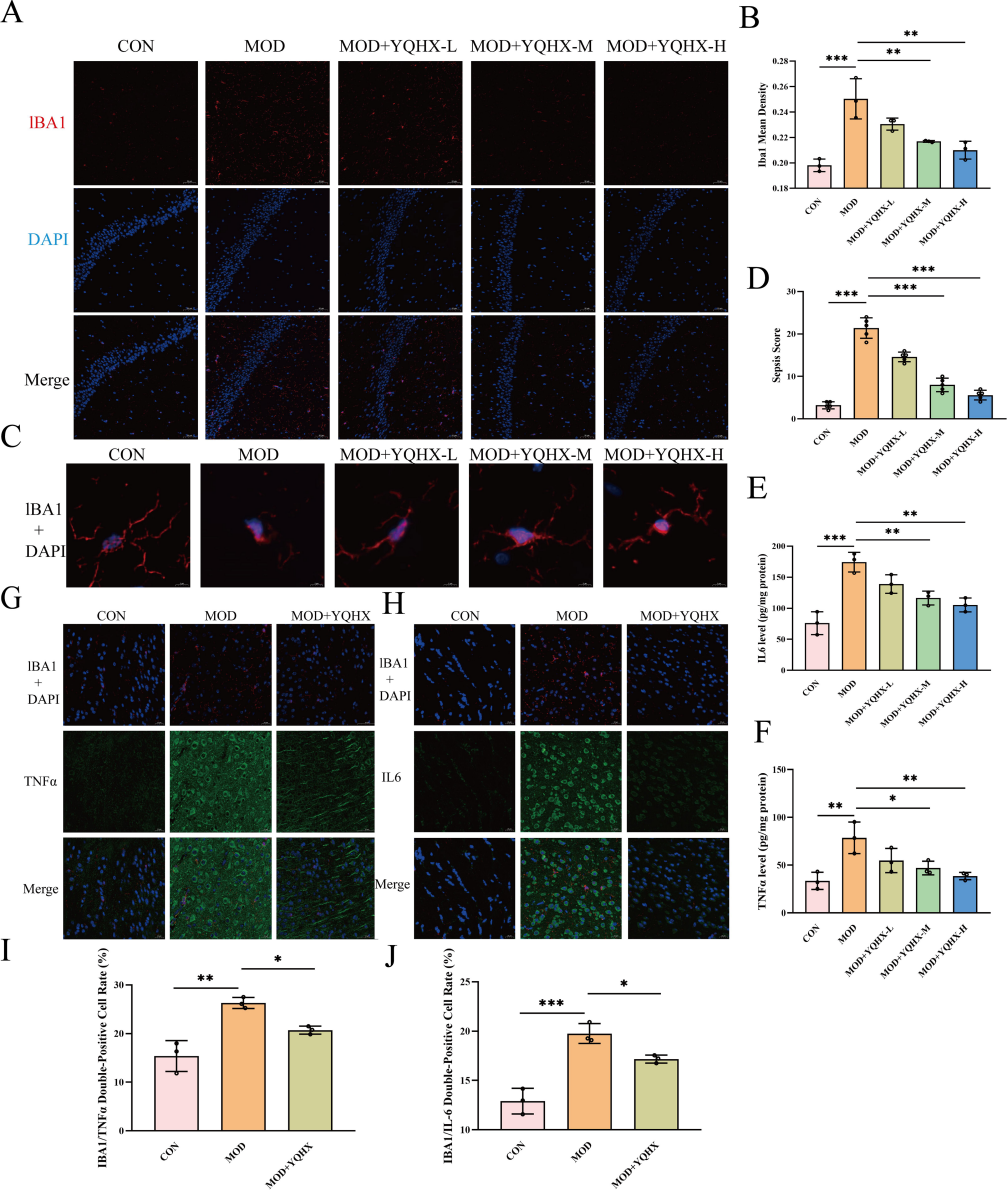
YQHXP attenuates LPS-induced inflammation and neuronal injury in hippocampal CA1. **(A)** Iba1 immunofluorescence in Control, Model, and YQHXP Low/Medium/High groups (representative fields). **(B)** Statistical plot of Iba1 fluorescence intensity: Model > Control; YQHXP Medium/High < Model (n=3). **(C)** Representative microglial morphology: Model shows enlarged soma with shorter and fewer processes; YQHXP Medium/High partially restores a more ramified appearance. **(D)** Statistical plot of sepsis scores: Model > Control; YQHXP Medium/High lower than Model(n=3)**. (E–F)** Statistical plots of hippocampal cytokines by ELISA: **(E)** TNFα and **(F)** IL-6. Model values exceed Control; YQHXP Medium/High reduce both versus Model(n=3). **(G–J)** Double-immunofluorescence for cytokine–microglia colocalization. **(G)** TNFα/Iba1 images with **(I)** corresponding statistical plot of double-positive cell fraction. **(H)** IL-6/Iba1 images with **(J)** corresponding statistical plot. Model shows increased colocalization; YQHXP reduces it(n=3). *P < 0.05 relative to the model group; **P < 0.01, ***P < 0.001 relative to the model group.

To evaluate whether YQHXP counteracts LPS-evoked neuroinflammation and neuronal injury, we assessed microglial activation, sepsis severity, cytokine levels, and cytokine–microglia colocalization in hippocampal CA1 (**Figure 2**). Model mice showed higher Iba1 immunofluorescence than Controls, whereas medium and high doses of YQHXP reduced Iba1 intensity; the low dose had no evident effect (**Figures 2A, B**). The sepsis score was elevated in Model mice and was lowered by medium and high YQHXP (**Figure 2C**). Morphologically, LPS yielded microglia with enlarged somata, shortened processes, and fewer branches; these features improved with medium and high YQHXP (**Figure 2D**). ELISA of hippocampal homogenates showed increased TNF-α and IL-6 in the Model group, both of which were reduced by medium and high YQHXP (**Figures 2E, F**). Because the low dose was not significant and the medium and high doses produced comparable improvements, the medium dose was used for subsequent *in vivo* studies. Dual-immunofluorescence further revealed increased TNF-α/Iba1 and IL-6/Iba1 double-positive fractions in Model mice versus Controls, both of which were decreased by YQHXP (quantified in **Figures 2I, J**; representative images in **Figures 2G, H**).

### 3.3 YQHXP plasma-absorbed constituents and network-pharmacology–based prediction of targets and pathways in SAE

**Figure 3:**
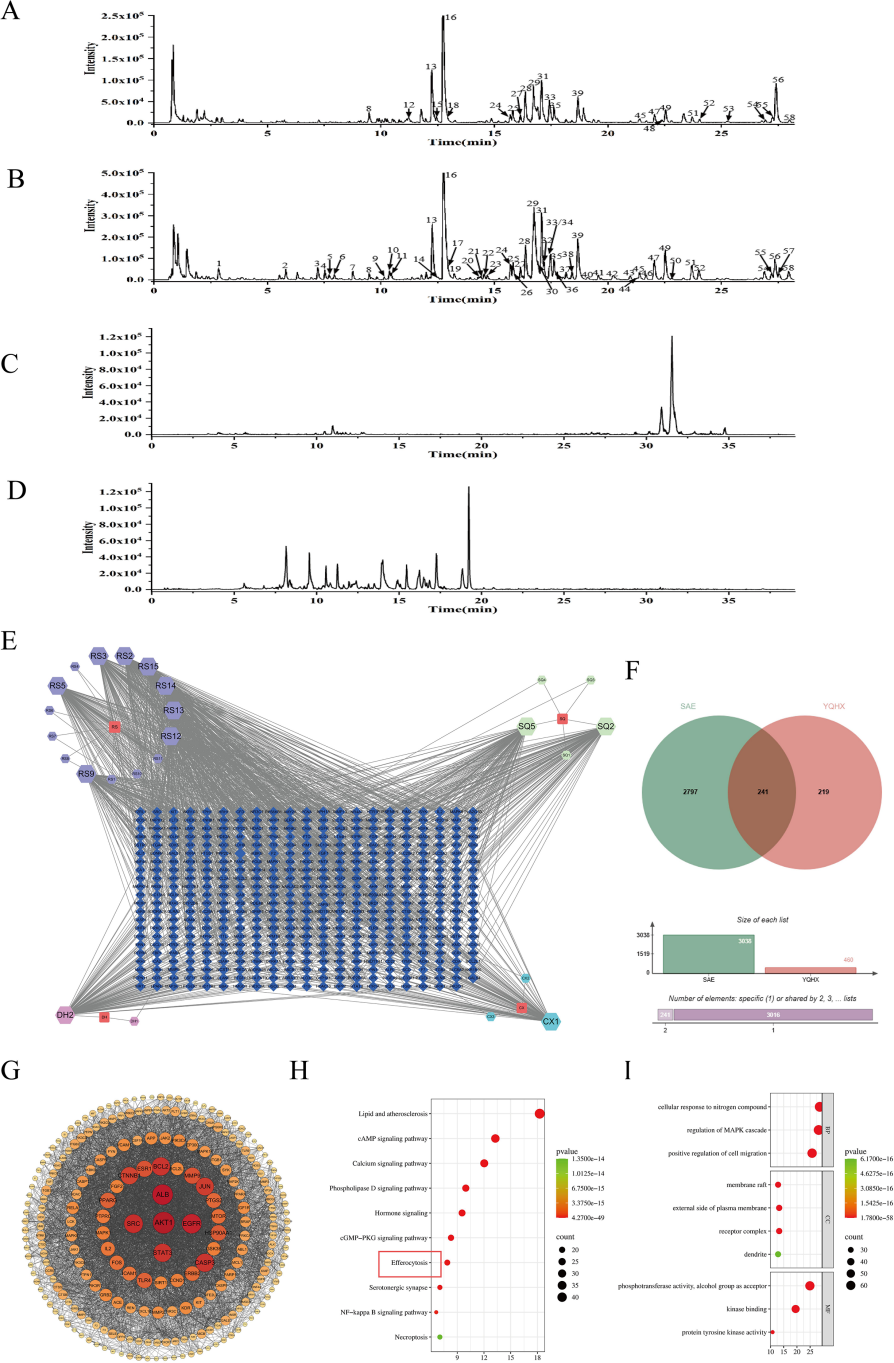
Plasma-absorbed constituents of YQHXP and network-pharmacology predictions for SAE. **(A–B)** UPLC–HRMS base-peak chromatograms (BPCs) of the YQHXP preparation in positive **(A)** and negative **(B)** ion modes. **(C–D)** Extracted-ion chromatograms (EICs) of serum after YQHXP administration in positive **(C)** and negative **(D)** ion modes, showing 25 circulating prototype compounds (**Table 1**). **(E)** Compound–target network linking the 25 prototypes to their predicted protein targets. **(F)** Venn diagram intersecting 460 predicted drug targets with 3,038 SAE-related targets, yielding 241 common targets. **(G)** Protein–protein interaction (PPI) network of the 241 common targets. **(H–I)** Enrichment analyses of the 241 common targets: **(H)** KEGG pathway bubble plot (bubble size reflects mapped-gene count; color encodes P value; includes efferocytosis), **(I)** GO enrichment.

UPLC–HRMS profiling of YQHXP generated base peak chromatograms (BPCs) in positive and negative ion modes (**Figures 3A, B**). In total, 58 constituents were annotated in the preparation (**Supplementary Table 1**, **Supplementary Figures S2**). After oral dosing, extracted ion chromatograms (EICs) from rat serum identified 25 circulating prototype compounds (**Figures 3C, D**), summarized with retention times and accurate masses in **Table 1**.

**Table 1:** Initial identification of compounds into the blood.

No.	Name of prototype	Retention time (min)	Adducts	Experimental m/z	Theoretical m/z	Mass error (ppm)
P1	Ferulic acid	10.56	[M-H]-	193.0512	193.0506	3.1
P2	Rhein-8-O-β-D-glucopyranoside	10.57	[M-H]-	445.0786	445.0776	2.2
P3	Notoginsenoside R1	12.64	[M^+^FA-H]-	977.5317	977.5321	-0.4
P4	Senkyunolide I	12.74	[M-H2O^+^H]^+^	207.1016	207.1016	0.0
P5	Ginsenoside Rg1	13.15	[M^+^FA-H]-	845.4878	845.4904	-3.1
P6	Ginsenoside Re	13.16	[M^+^FA-H]-	991.5426	991.5483	-5.7
P7	Senkyunolide H	13.29	[M-H2O^+^H]^+^	207.1018	207.1016	1.0
P8	Ginsenoside Ra3	16.24	[M ^+^ 2FA-2H]2-	665.3216	665.3202	2.1
P9	Notoginsenoside Fa	16.68	[M ^+^ 2FA-2H]2-	665.3195	665.3202	-1.1
P10	Ginsenoside Rb1	17.27	[M^+^FA-H]-	1153.6082	1153.6011	6.2
P11	Ginsenoside F1	17.59	[M^+^FA-H]-	683.4369	683.4376	-1.0
P12	Ginsenoside Rc	17.73	[M^+^FA-H]-	1123.5947	1123.5906	3.6
P13	Ginsenoside Ro	17.96	[M-H]-	955.4892	955.4908	-1.7
P14	Ginsenoside Rh1	17.98	[M^+^FA-H]-	683.4391	683.4376	2.2
P15	Ginsenoside Rb2	18.25	[M^+^FA-H]-	1123.5895	1123.5906	-1.0
P16	Ginsenoside Rb3	18.57	[M^+^FA-H]-	1123.5925	1123.5906	1.7
P17	Quinquenoside R1	18.72	[M^+^FA-H]-	1195.6111	1195.6117	-0.5
P18	Ginsenoside Rd	19.23	[M^+^FA-H]-	991.5503	991.5483	2.0
P19	Gypenoside XVII	20.14	[M^+^FA-H]-	991.5450	991.5483	-3.3
P20	Rhein	20.74	[M-H]-	283.0246	283.0248	-0.7
P21	Ginsenoside Rk3	22.59	[M^+^FA-H]-	665.4261	665.4270	-1.4
P22	20(S)-Ginsenoside Rg3	22.89	[M^+^FA-H]-	829.4963	829.4955	1.0
P23	Ginsenoside Rh4	23.06	[M^+^FA-H]-	665.4244	665.4270	-3.9
P24	Zingibroside R1	23.34	[M-H]-	793.4357	793.4380	-2.9
P25	20(R)-Ginsenoside Rg3	24.25	[M^+^FA-H]-	829.4955	829.4955	0.0

For network pharmacology, the 25 prototypes were submitted to SwissTargetPrediction (Homo sapiens), yielding 460 putative targets. SAE-related genes (n = 3,038) were compiled from GeneCards and OMIM. Intersecting these sets produced 241 shared targets (**Figure 3F**), visualized as a compound–target network (**Figure 3E**) and a STRING protein–protein interaction map (**Figure 3G**). KEGG enrichment of the 241 targets highlighted lipid and atherosclerosis, cAMP signaling, calcium signaling, phospholipase D signaling, hormone signaling, cGMP–PKG signaling, serotonergic synapse, NF-κB signaling, necroptosis, and efferocytosis (**Figure 3H**). GO analysis (BP/CC/MF) indicated enrichment for regulation of MAPK cascade, positive regulation of cell migration, membrane raft, external side of plasma membrane, receptor complex, dendrite, kinase binding, and protein tyrosine kinase activity (**Figure 3I**). Guided by these results and prior literature, efferocytosis signaling were prioritized for subsequent mechanistic validation in the SAE context.

### 3.4 Transcriptomics integrated with network pharmacology identifies key targets of YQHXP in SAE

**Figure 4:**
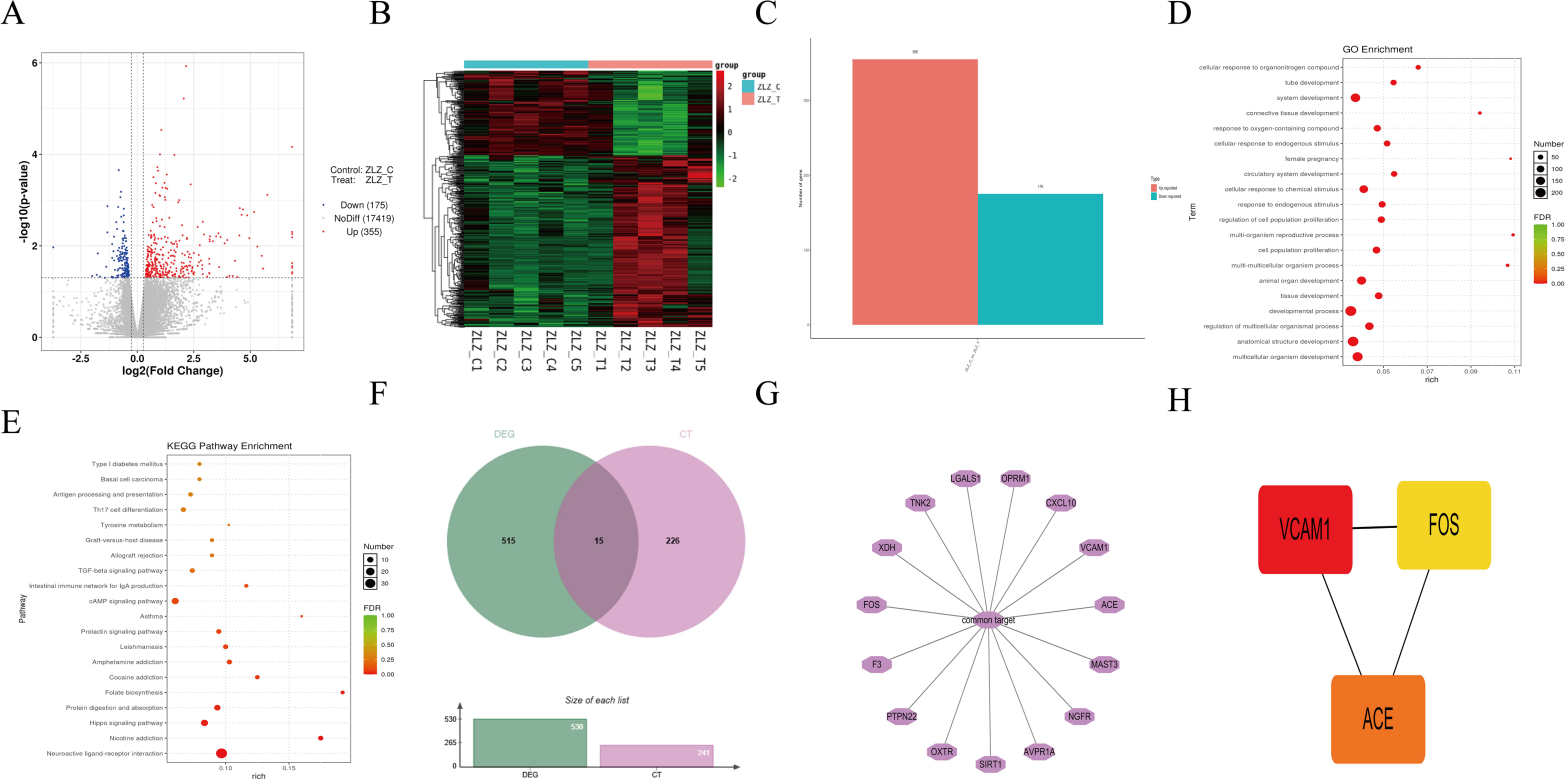
Transcriptomics integrated with network pharmacology reveals key targets of YQHXP in SAE. **(A)** Volcano plot of DEGs between Model and YQHXP hippocampi (blue, downregulated; red, upregulated). **(B)** Heatmap of significant DEGs; green-to-red color scale indicates low-to-high expression. **(C)** Bar chart summarizing DEGs: 355 upregulated and 175 downregulated genes. (D) KEGG pathway enrichment based on DEGs. **(E)** GO enrichment (BP/CC/MF) for the same DEG set. **(F–G)** Venn diagram intersecting DEGs with compound–disease common targets (CT), identifying 15 overlapping genes (key targets). **(H)** cytoHubba-based ranking of the 15 key targets, with VCAM1 prioritized as the core hub.

RNA-seq of hippocampal tissue from Model and YQHXP–treated mice identified 530 DEGs. A volcano plot illustrates the distribution of significant changes (**Figure 4A**; blue, downregulated; red, upregulated), and a heatmap shows groupwise expression patterns with clear sample separation (**Figure 4B**; green-to-red denotes low-to-high expression). In total, 355 genes were upregulated and 175 were downregulated (**Figure 4C**). Functional annotation of these DEGs is summarized by KEGG pathway enrichment (**Figure 4D**) and GO enrichment across BP/CC/MF categories (**Figure 4E**).

To pinpoint mechanistic mediators of YQHXP in SAE, DEGs were intersected with the previously derived compound–disease CTs. The Venn analysis yielded 15 overlapping genes, designated as candidate key targets (**Figure 4G**). Prioritization using cytoHubba highlighted VCAM1 as the top-ranked hub within this set (**Figure 4H**).

### 3.5 YQHXP–containing serum suppresses pro-inflammatory cytokines, limits apoptosis, and enhances efferocytosis in BV2 microglia

**Figure 5:**
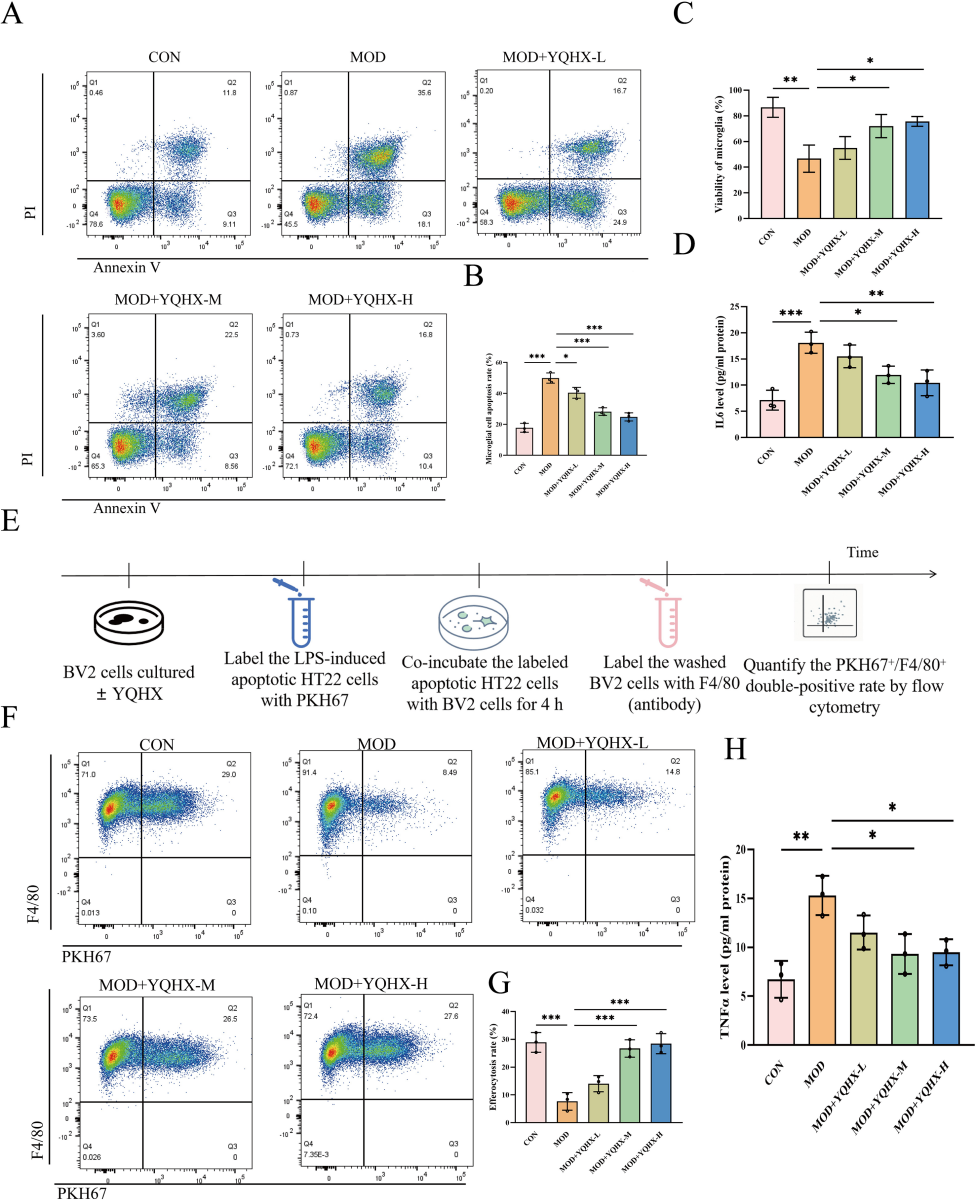
YQHXP–containing serum limits LPS injury and promotes efferocytosis in BV2 microglia. **(A)** Representative flow-cytometry plots of BV2 apoptosis across groups. **(B)** Statistical plot of apoptotic fraction(n=3). **(C)** Statistical plot of CCK-8 cell viability(n=3). **(D, H)** Statistical plots of ELISA-measured cytokines in supernatants: **(D)** TNFα(n=3); **(H)** IL-6(n=3). **(E)** Schematic of the efferocytosis co-culture: BV2 (Control/Model/low/medium/high); LPS to all but Control; apoptotic HT22 labeled with PKH67; 4 h co-incubation; BV2 labeled with F4/80; flow cytometry to quantify PKH67^+^/F4/80^+^ BV2 cells. **(F)** Representative flow-cytometry readouts of efferocytosis. **(G)** Statistical plot of efferocytosis rate (PKH67^+^/F4/80^+^ double-positive rate, n=3). *P < 0.05 relative to the model group; **P < 0.01, ***P < 0.001 relative to the model group.

In an *in-vitro* SAE model, BV2 microglia were allocated to five groups: Control, LPS Model, and YQHXP–containing serum at low, medium, or high concentration. Flow cytometry showed that LPS markedly increased BV2 apoptosis relative to Control, whereas medium and high YQHXP–containing serum reduced the apoptotic fraction; the low concentration had no evident effect (**Figures 5A, B**). Consistently, CCK-8 assays indicated that LPS suppressed cell viability, which was restored by medium and high YQHXP–containing serum but not by the low concentration (**Figure 5C**). ELISA of culture supernatants further revealed LPS-induced increases in TNF-α and IL-6; both cytokines were lowered by medium and high YQHXP–containing serum, with no clear change at the low concentration (**Figure 5D, 5H**).

To assess microglial efferocytosis, PKH67-labeled apoptotic HT22 neurons were co-cultured with BV2 cells for 4 h; BV2 were stained with F4/80, and the PKH67^+^/F4/80^+^ double-positive fraction was quantified by flow cytometry as the efferocytosis rate (workflow in **Figure 5E**). LPS impaired efferocytosis compared with Control, whereas medium and high YQHXP–containing serum increased the double-positive fraction versus the Model group (**Figures 5F, G**; **Supplementary Figure S3**). These data support enhanced microglial efferocytosis as a plausible mechanism by which YQHXP mitigates SAE-relevant pathology.

### 3.6 YQHXP modulates a VCAM1-linked efferocytosis program (C1QB/MERTK) in BV2 microglia and hippocampus

**Figure 6:**
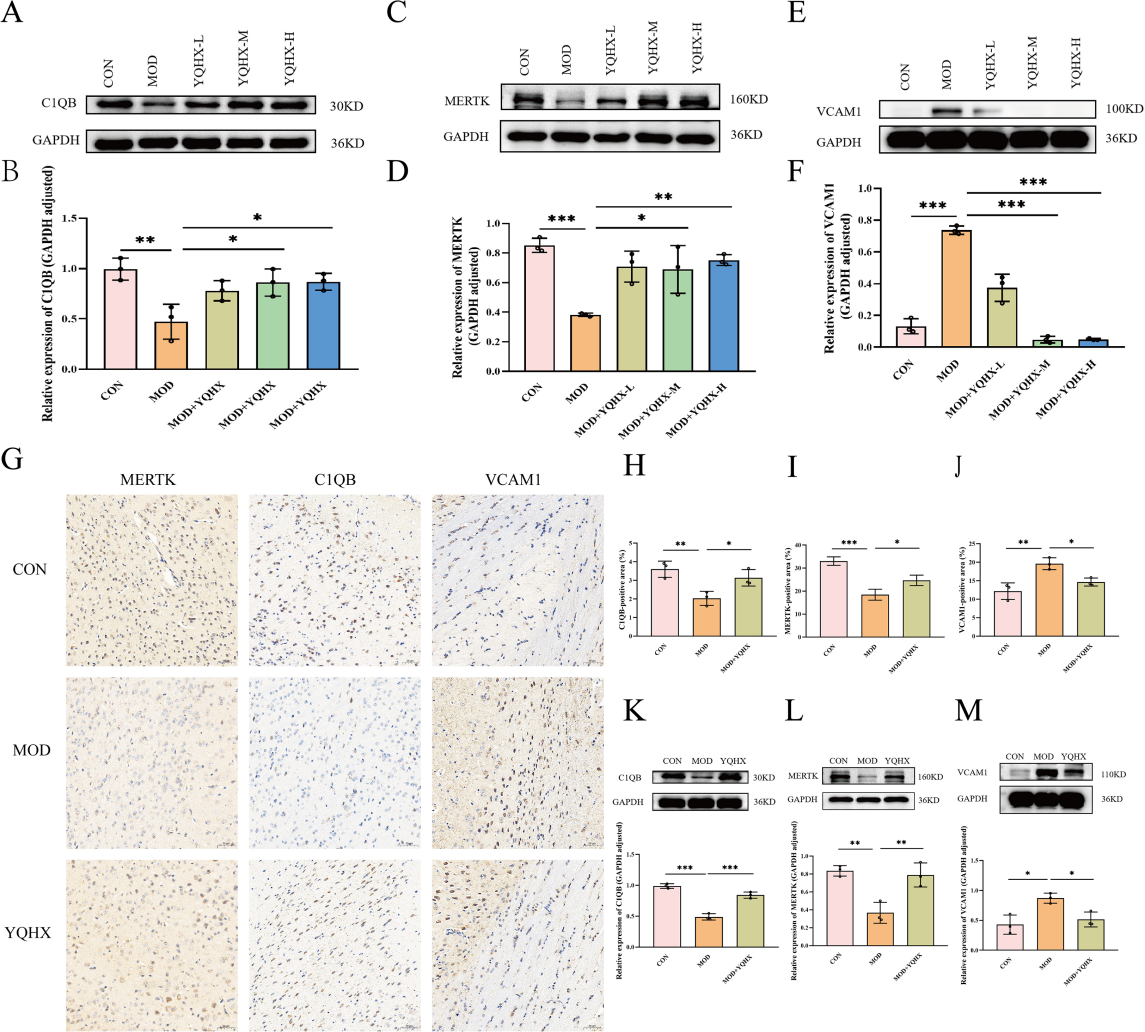
YQHXP restores a pro-efferocytosis signature (C1QB/MERTK) and suppresses VCAM1 in vitro and in vivo. **(A)** Representative in-vitro expression of C1QB in BV2 cells across groups (Control, Model/LPS, YQHXP–containing serum Low/Medium/High). **(B)** Statistical plot for C1QB(n=3). **(C)** Representative in-vitro expression of MERTK. **(D)** Statistical plot for MERTK(n=3). **(E)** Representative in-vitro expression of VCAM1. **(F)** Statistical plot for VCAM1(n=3). **(G)** Hippocampal immunohistochemistry for C1QB, MERTK, and VCAM1 in Control, Model, and YQHXP groups (representative fields). **(H–J)** Statistical plots for IHC quantification: **(H)** C1QB(n=3), **(I)** MERTK(n=3), **(J)** VCAM1(n=3); YQHXP reverses Model-induced changes. **(K–M)** Hippocampal Western blots with corresponding statistical plots: **(K)** C1QB(n=3), **(L)** MERTK(n=3), **(M)** VCAM1(n=3); results mirror the in-vitro patterns. *P < 0.05 relative to the model group; **P < 0.01, ***P < 0.001 relative to the model group.

In LPS-treated BV2 cells (Model), C1QB and MERTK were decreased relative to Control, whereas VCAM-1 was increased. YQHXP–containing serum at medium and high concentrations increased C1QB (**Figures 6A, B**) and MERTK (**Figures 6C, D**) and decreased VCAM-1 (**Figures 6E, F**) versus Model. Given the consistent improvements at the medium concentration across assays, this dose was selected for subsequent *in-vitro* experiments.

In mice, hippocampal immunohistochemistry showed reduced C1QB and MERTK and elevated VCAM-1 in Model versus Control; YQHXP reversed these changes (representative images, **Figure 6G**; quantification, **Figures 6H–J**). Western blots of hippocampal tissue corroborated the immunohistochemical findings, with C1QB and MERTK restored and VCAM-1 suppressed after YQHXP treatment (**Figures 6K–M**). Taken together with Sections 3.3–3.5, the concurrent upregulation of C1QB/MERTK and suppression of VCAM-1 supports a mechanism whereby YQHXP mitigates SAE via a VCAM-1–linked efferocytosis pathway.

### 3.7 Pharmacological perturbation supports a VCAM1-linked efferocytosis and anti-inflammatory mechanism for YQHXP in SAE

**Figure 7:**
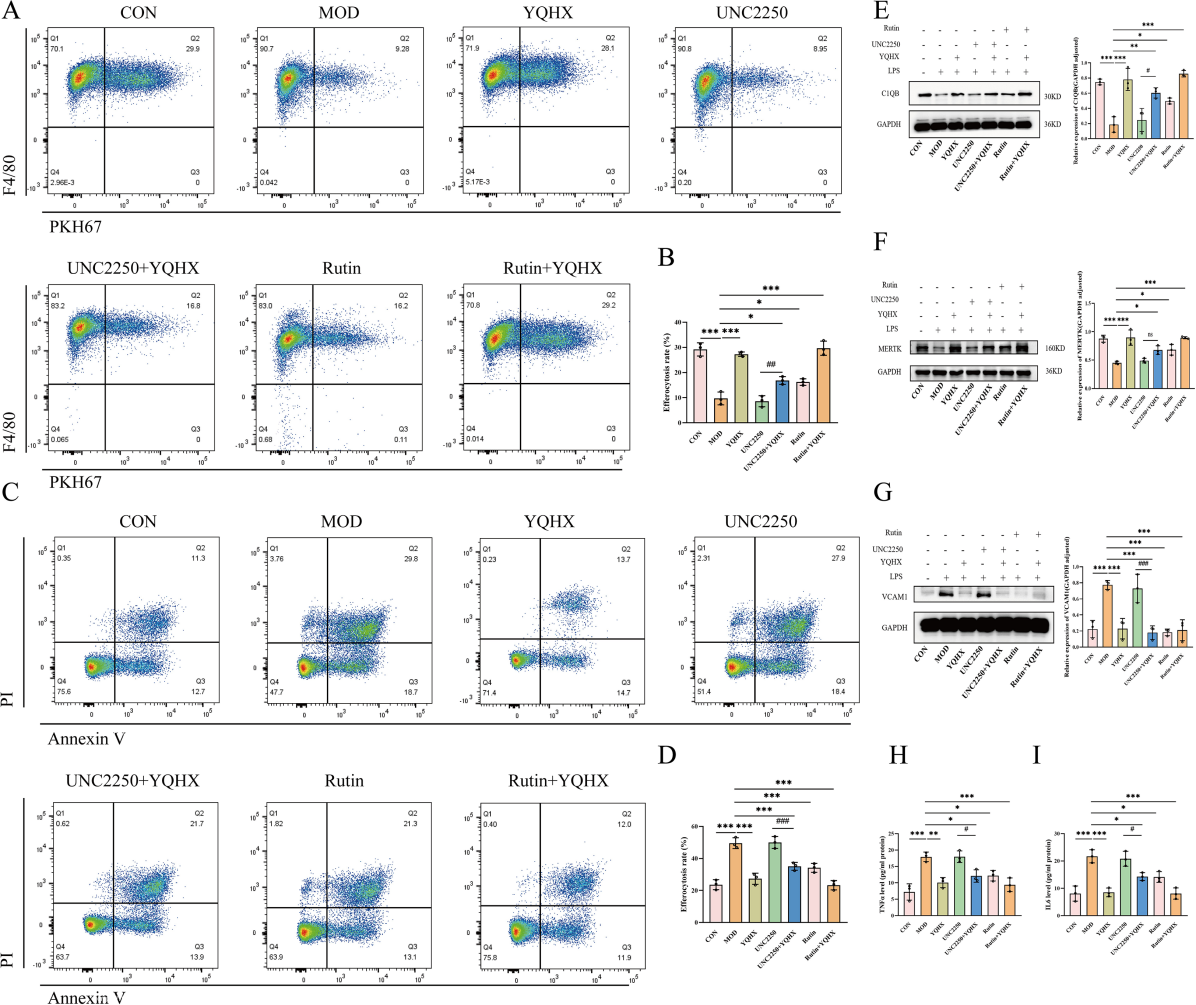
Pharmacological modulation of MERTK and VCAM1 implicates a VCAM1-linked efferocytosis and anti-inflammatory mechanism for YQHXP. **(A)** Flow cytometry analysis of PKH67^+^/F4/80^+^ double-positive cells in BV2–HT22 co-culture across seven groups. **(B)** Quantification of efferocytosis rate(n=3). **(C)** Flow cytometry plots of apoptotic BV2 cells (Annexin V/PI staining). **(D)** Quantification of apoptosis rate(n=3). **(E)** Western blot and quantification of C1QB protein expression(n=3). **(F)** Western blot and quantification of MERTK protein expression(n=3). **(G)** Western blot and quantification of VCAM1 protein expression(n=3). **(H)** TNF-α levels in BV2 supernatant measured by ELISA(n=3). IL-6 levels in BV2 supernatant measured by ELISA(n=3). *P < 0.05 relative to the model group; **P < 0.01, ***P < 0.001 relative to the model group. ^#^P < 0.05 relative to the UNC2250 group; ^##^P < 0.01 relative to the UNC2250 group; ^###^P < 0.001 relative to the UNC2250 group.

To test whether YQHXP acts through VCAM-1–linked efferocytosis, BV2 microglia were assigned to seven groups: Control, Model (LPS), YQHXP, UNC2250, UNC2250^+^YQHXP, rutin, and rutin^+^YQHXP (LPS applied to all except Control). In a BV2–HT22 co-culture assay, flow cytometry showed that LPS reduced BV2 efferocytosis, quantified as the PKH67^+^/F4/80^+^ double-positive fraction. YQHXP, UNC2250^+^YQHXP, rutin, and rutin^+^YQHXP each increased efferocytosis versus Model; moreover, UNC2250^+^YQHXP partially restored efferocytosis relative to UNC2250 alone (**Figures 7A, B**). Apoptosis analysis indicated that LPS markedly increased BV2 apoptosis compared with Control, whereas YQHXP, UNC2250^+^YQHXP, rutin, and rutin^+^YQHXP reduced apoptosis versus Model; co-treatment with YQHXP also lowered apoptosis relative to UNC2250 alone (**Figures 7C, D**).

At the protein level, C1QB (**Figure 7E**) and MERTK (**Figure 7F**) were increased in the YQHXP, UNC2250^+^YQHXP, rutin, and rutin^+^YQHXP groups versus Model; C1QB in UNC2250^+^YQHXP groups also exceeded UNC2250 alone. For MERTK, only rutin^+^YQHXP was higher than UNC2250, whereas UNC2250^+^YQHXP remained comparable to UNC2250, consistent with strong MERTK inhibition during co-treatment. By contrast, VCAM-1—elevated by LPS—was reduced by YQHXP, UNC2250^+^YQHXP, rutin, and rutin^+^YQHXP versus Model, and each of the latter three was lower than UNC2250 alone (**Figure 7G**). ELISA of culture supernatants confirmed higher TNF-α and IL-6 in the Model group; YQHXP, UNC2250^+^YQHXP, rutin, and rutin^+^YQHXP each reduced both cytokines versus Model, and YQHXP co-treatment further decreased cytokine release relative to UNC2250 alone (**Figures 7H, I**).

Taken together, these molecular patterns and the efferocytosis measurements support a mechanism in which YQHXP mitigates SAE by enhancing efferocytosis through a VCAM-1–linked pathway while limiting inflammatory cytokine production.

### 3.8 Molecular docking, MD simulations, and SPR substantiate VCAM-1 engagement by YQHXP prototypes

**Figure 8:**
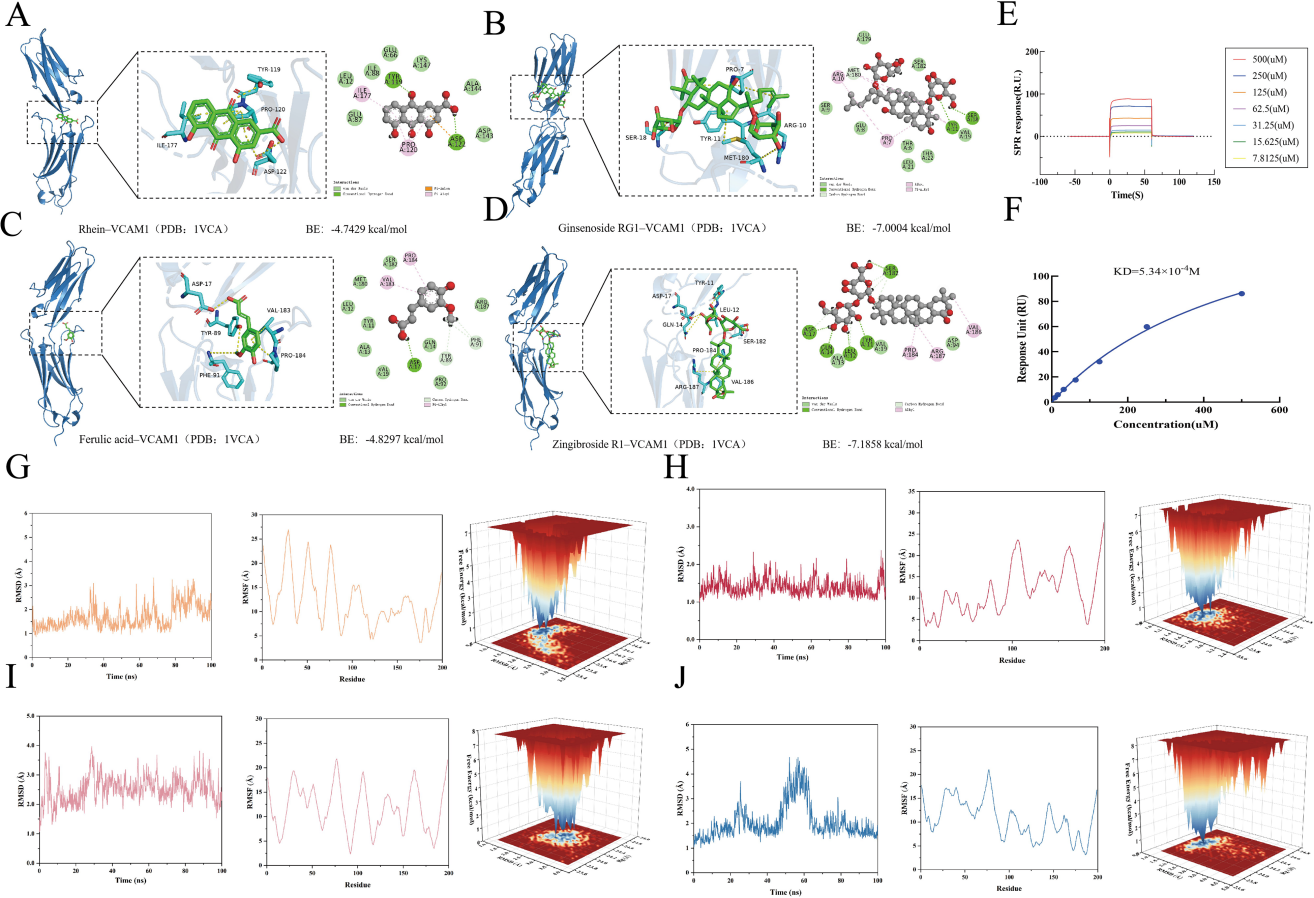
Docking, molecular dynamics, and SPR support direct interactions between VCAM-1 and YQHXP prototype constituents. **(A)** Rhein–VCAM-1 docking pose (MOE 2019). **(B)** Ginsenoside Rg1–VCAM-1 docking pose. **(C)** Ferulic acid–VCAM-1 docking pose. **(D)** Zingibroside R1–VCAM-1 docking pose. Docking scores (kcal/mol): Rg1 −7.0004, Zingibroside R1 −7.1858, Ferulic acid −4.8297, Rhein −4.7429. **(E)** SPR sensorgrams for Ginsenoside Rg1 binding to immobilized human VCAM-1 across a concentration series. **(F)** Steady-state concentration–response fit, yielding KD = 5.34×10-^4^M. **(G–J)** 100-ns MD analyses of ligand–VCAM-1 complexes: RMSD, RMSF, and 3D free-energy landscapes (FEL). Complexes reach equilibrium by ~30 ns for Rg1, Ferulic acid, and Rhein, and by ~60 ns for Zingibroside R1. Mean RMSD (A): Rg1 1.40, Zingibroside R1 2.05, Ferulic acid 2.52, Rhein 1.69. RMSF ranges (A) and means: Rg1 3.09–27.70 (11.71), Zingibroside R1 3.12–21.05 (10.68), Ferulic acid 2.32–21.84 (11.73), Rhein 3.60–26.94 (12.16). Additional MD readouts (radius of gyration, hydrogen-bond counts, and 2D FEL maps) are provided in Supplementary Figures.

To evaluate whether plasma-exposed prototypes of YQHXP engage VCAM-1, we first performed docking in MOE (2019). All four ligands showed favorable scores with VCAM-1—ginsenoside Rg1 (−7.0004 kcal/mol), zingibroside R1 (−7.1858 kcal/mol), ferulic acid (−4.8297 kcal/mol), and rhein (−4.7429 kcal/mol)—placing the compounds in surface grooves with complementary hydrogen-bonding and hydrophobic contacts (**Figures 8A–D**).

Complex stability was then assessed by 100-ns MD simulations. RMSD traces plateaued at ~30 ns for Rg1–VCAM-1, ferulic acid–VCAM-1, and rhein–VCAM-1, and at ~60 ns for zingibroside R1–VCAM-1. Mean backbone RMSDs were 1.40, 2.05, 2.52, and 1.69 Å, respectively, consistent with stable trajectories. Residue-wise RMSF indicated modest local flexibility and subtle conformational adjustments upon binding, and free-energy landscape maps revealed one or more low-energy basins for each complex, supporting preferred, kinetically accessible bound states (**Figures 8G–J**; **Supplementary Figure S5–8**).

Finally, surface plasmon resonance quantified protein–ligand affinity. Under a 1:1 steady-state model, ginsenoside Rg1 bound VCAM-1 with KD = 5.34 × 10^-4^M, corroborating docking/MD predictions and providing a physical basis for endothelial VCAM-1 modulation by YQHXP constituents (**Figures 8E, F**).

## 4 Discussion

This study suggests that YQHXP mitigates sepsis-associated encephalopathy by engaging a VCAM-1–linked efferocytosis program in microglia. In LPS-challenged mice, YQHXP improved performance in the Morris water maze and increased center-zone exploration in the open-field test, accompanied by lower hippocampal TNF-α and IL-6 and reduced microglial activation (**Figures 1**, **2**). Integrated network pharmacology and hippocampal transcriptomics highlighted efferocytosis and nominated VCAM-1 as actionable readouts. Consistently, YQHXP downregulated VCAM-1, upregulated C1QB and MERTK, and increased the PKH67^+^/F4/80^+^ double-positive fraction *in vitro* assay that reports uptake of apoptotic neurons (**Figures 3–6**). Pharmacological perturbation aligned with this dual-node model: the MERTK inhibitor UNC2250 (31)curtailed efferocytosis, whereas VCAM-1 suppression by rutin (32) enhanced efferocytosis and partially restored clearance even when MERTK was constrained, supporting an upstream gating role for VCAM-1 within the efferocytic axis (**Figure 7**). Taken together, these data indicate that YQHXP dampens endothelial VCAM-1 signaling while strengthening microglial efferocytosis, and that these mechanisms act in concert to reduce inflammatory burden and improve behavioral outcomes.

Mechanistically, C1QB and MERTK link molecular modulation to efferocytic capacity (33, 34). C1QB, a core subunit of the C1q complex, opsonizes phosphatidylserine-exposing apoptotic cells to stabilize recognition and promote engulfment; C1q signaling also biases microglia toward pro-resolving states, limits danger-associated molecular pattern release, and helps avert secondary necrosis (35–37). MERTK, a TAM-family receptor, senses “eat-me” cues on apoptotic targets and couples recognition to internalization, while downstream signaling dampens pro-inflammatory drive and coordinates the cytoskeletal remodeling required for efficient uptake (38, 39). In this context, YQHXP–associated upregulation of C1QB and MERTK, together with increased efferocytosis, provides a plausible cellular route to lowering cytokine burden, preventing accumulation of toxic remnants, and preserving neural function (15, 40).

Molecular evidence strengthens the plausibility of this framework. Docking and 100-ns molecular dynamics predicted favorable interactions between VCAM-1 and several plasma-exposed prototypes of YQHXP (ginsenoside Rg1, zingibroside R1, ferulic acid, rhein). Surface plasmon resonance confirmed direct VCAM-1 binding by ginsenoside Rg1 in the sub-millimolar range, corroborating the computational predictions and providing a physical basis for endothelial target engagement. Prior mechanistic and therapeutic investigations in SAE have primarily emphasized systemic anti-inflammatory interventions, supportive hemodynamic management (including fluid resuscitation), and sedation strategies (41). As the BBB is a critical determinant of central nervous system homeostasis and disease progression, improving the delivery of therapeutics across the BBB to the brain remains a major translational challenge (42). In this context, brain endothelial VCAM-1 emerges as a tractable upstream node at the neurovascular interface; its modulation is coupled to downstream reinforcement of microglial efferocytosis through the C1QB/MERTK axis, accompanied by measurable behavioral benefits.Rather than relying solely on cytokine suppression, YQHXP may modulate the inflammatory trajectory by coupling endothelial deactivation with enhanced clearance of apoptotic debris. Although docking and MD are hypothesis-generating and the measured affinity is moderate, convergence across computational, biophysical, and biological layers supports VCAM-1 as a pharmacodynamic marker within an efferocytosis-centered framework.

Beyond suggesting endothelial target engagement, the plasma-exposed prototypes defined by UPLC–HRMS provide a chemically plausible basis for the multi-level phenotype elicited by YQHXP. Ginsenoside Rg1 has been reported to attenuate systemic inflammatory injury by suppressing pro-inflammatory signaling and oxidative stress in endotoxin- or sepsis-related experimental settings, consistent with an overall shift toward inflammatory resolution (43, 44). Ferulic acid, a phenylpropanoid with well-characterized antioxidant activity, has been shown to dampen cytokine-driven endothelial activation and reduce adhesion-molecule expression in inflammatory models, aligning with our observation that YQHXP downregulates VCAM-1 (45). Rhein, an anthraquinone prototype, has been reported to modulate innate-immune pathways such as TLR4/NF-κB and NLRP3 inflammasome signaling and to mitigate inflammatory amplification cascades, which could complement an efferocytosis-centered mechanism by lowering mediator burden that otherwise compromises phagocyte function (46, 47). Zingibroside R1 has likewise been associated with anti-inflammatory and anti-oxidative effects in LPS-linked injury models, providing additional coverage across stress-response pathways relevant to sepsis (48). Importantly, these prototypes should not be interpreted as single-agent surrogates for the formula; rather, their co-exposure after oral dosing is consistent with the multi-component, multi-target pharmacology typical of herbal prescriptions (49, 50). Collectively, this evidence supports a complementary mode of action in which endothelial deactivation and reinforcement of microglial resolution programs may act in concert to curb cytokine burden, limit accumulation of apoptotic debris, and preserve neurovascular function.

This study has limitations. First, while the LPS model reproduces key features of neuroinflammation, it does not capture the heterogeneity of polymicrobial sepsis—hemodynamic instability, pathogen diversity, and multi-organ crosstalk (51, 52). Follow-up work should validate these findings in cecal ligation and puncture and related infection models, and include female and aged animals to test external validity. Second, drug-containing serum reflects plasma-exposed prototypes after oral dosing but cannot substitute for human pharmacokinetics, metabolism, and tissue distribution. Third, although docking, 100-ns MD, and SPR support VCAM-1 as a direct target of ginsenoside Rg1, the relevance of this interaction to SAE pathobiology requires further substantiation. Finally, YQHXP is a multi-component, multi-target formulation; the present study focused on VCAM-1 and efferocytosis, and additional mechanisms warrant systematic investigation.

Within these bounds, our findings support a VCAM-1–linked efferocytosis axis as a tractable translational direction for SAE and clarify how it may be operationalized in future development. From a clinical pharmacology perspective, soluble VCAM-1 (sVCAM-1) is attractive as a pharmacodynamic readout because it can be measured longitudinally in peripheral blood using routine immunoassays, enabling time-resolved assessment of endothelial activation during sepsis and septic encephalopathy (53–56). In early-phase evaluation, serial sVCAM-1 trajectories could inform dose–response relationships and biological pathway modulation, particularly when interpreted alongside neurocognitive measures and inflammatory profiles, given the current limitations of single biomarkers for SAE in clinical practice (23). In parallel, efferocytosis-centered endpoints may provide proof-of-mechanism beyond cytokine suppression by capturing apoptotic-debris handling and resolution biology, including circulating DAMP-related signatures, extracellular vesicle–based markers, and, where feasible in translational settings, imaging- or tissue-based indices of debris clearance (57–61). Collectively, these measures would help distinguish an “endothelial deactivation - enhanced microglial clearance” phenotype from nonspecific anti-inflammatory effects and could guide subsequent outcome-focused studies.

## 5 Conclusion

YQHXP exerts significant neuroprotective effects against experimental SAE by deactivating VCAM-1–mediated endothelial signaling, which in turn enhances microglial efferocytosis; together with attenuation of hippocampal inflammation and apoptosis, this preserves neurovascular function and cognition. These findings provide fresh insight into the clinical relevance of YQHXP for SAE management.

## Data Availability

The data presented in the study are deposited in the NCBI Sequence Read Archive (SRA) repository, accession number PRJNA1441845.
