# Supplementary material for: YiQi-HuoXue prescription ameliorates LPS-induced sepsis-associated encephalopathy via VCAM-1–mediated microglial efferocytosis
Source: Front Immunol. 2026 Apr 1;17:1792688. doi: 10.3389/fimmu.2026.1792688 (PMC13079163; doi:10.3389/fimmu.2026.1792688)
Supplement: Supplementary file 1 [file Table1.docx]

### Supplementary Material: Western Blot Data

**Western blot bands of C1QB in BV2 cells (Control, Model, and YQHXP high, medium, and low groups)**

**Sample 1**

#### 1. Full Membrane Images


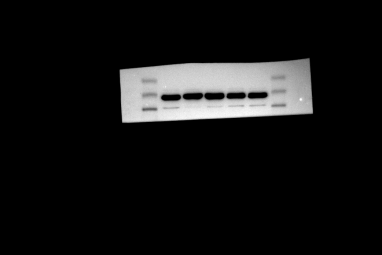


**Figure 1**: Full membrane image of C1QB and GAPDH.

#### 2. Cropped Target Protein Bands


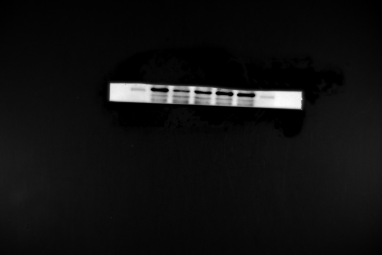


A


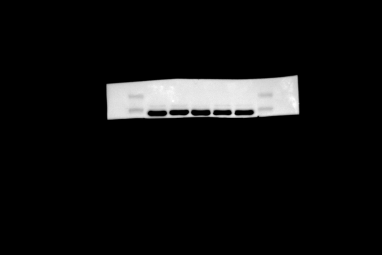


B

**Figure 2**: Cropped target protein bands from full membrane (as shown in Figure 1).

**A**. Target protein band for C1QB.

**B**. Cropped internal reference protein band (GAPDH).

**Sample 2**

#### 1. Full Membrane Images


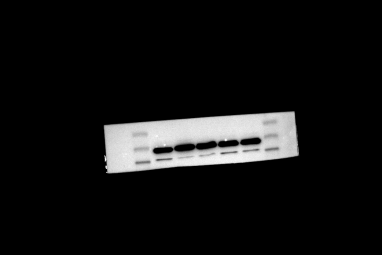


**Figure 3**: Full membrane image of C1QB and GAPDH.

#### 2. Cropped Target Protein Bands


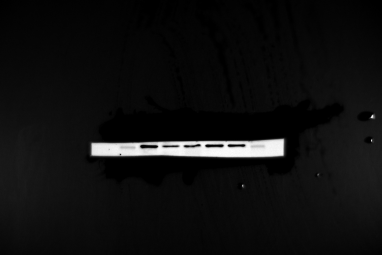


A


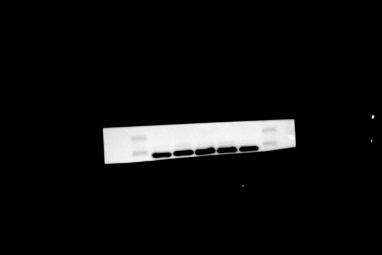


B

**Figure 4**: Cropped target protein bands from full membrane (as shown in Figure 3).

**A**. Target protein band for C1QB.

**B**. Cropped internal reference protein band (GAPDH).

**Sample 3**

#### 1. Full Membrane Images


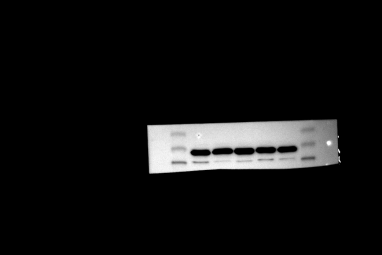


**Figure 5**: Full membrane image of C1QB and GAPDH.

#### 2. Cropped Target Protein Bands


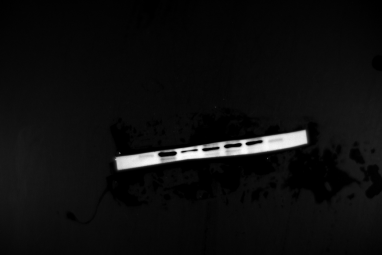


A


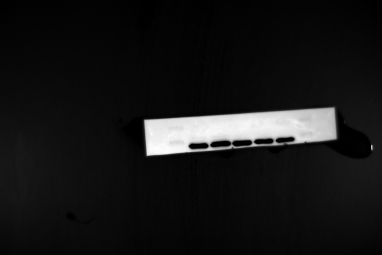


B

**Figure 6**: Cropped target protein bands from full membrane (as shown in Figure 5).

**A**. Target protein band for C1QB.

**B**. Cropped internal reference protein band (GAPDH).

| **Sample Description** | **Target Protein (Grayscale Value)** | | | | | **Internal Reference (Grayscale Value)** | | | | | **Normalized Value** | | | | |
| --- | --- | --- | --- | --- | --- | --- | --- | --- | --- | --- | --- | --- | --- | --- | --- |
|  | **CON** | **MOD** | **L-dose** | **M-dose** | **H-dose** | **CON** | **MOD** | **L-dose** | **M-dose** | **H-dose** | **CON** | **MOD** | **L-dose** | **M-dose** | **H-dose** |
| Sample 1 | 29717.61 | 20372.317 | 23524.388 | 28619.681 | 28599.803 | 33571.711 | 33024.903 | 34626.56 | 28740.368 | 30204.024 | 0.885197957 | 0.616877421 | 0.679374099 | 0.995800784 | 0.946887176 |
| Sample 2 | 29828.903 | 16244.267 | 18938.51 | 24375.024 | 21411.56 | 27021.489 | 31284.539 | 35721.782 | 33653.418 | 27398.489 | 1.103895607 | 0.519242652 | 0.781486891 | 0.724295642 | 0.781486891 |
| Sample 3 | 31354.51 | 9903.974 | 22431.388 | 25945.217 | 24410.418 | 31483.368 | 35517.004 | 33055.832 | 30026.418 | 27741.004 | 0.995907109 | 0.278851617 | 0.879939962 | 0.864079658 | 0.879939962 |

Table 1: Grayscale values of C1QB. bands analyzed using ImageJ.

**Western blot bands of VCAM1 in BV2 cells (Control, Model, and YQHXP high, medium, and low groups)**

**Sample 1**

#### 1. Full Membrane Images


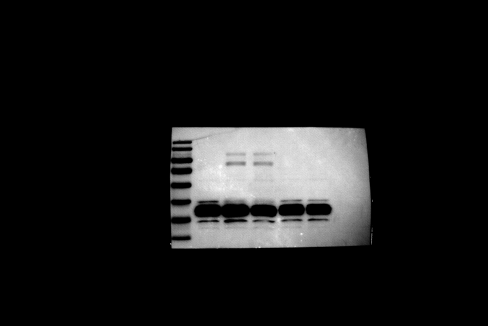


**Figure 7**: Full membrane image of VCAM1 and GAPDH.

#### 2. Cropped Target Protein Bands


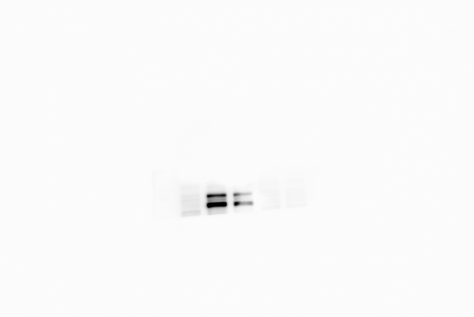


A


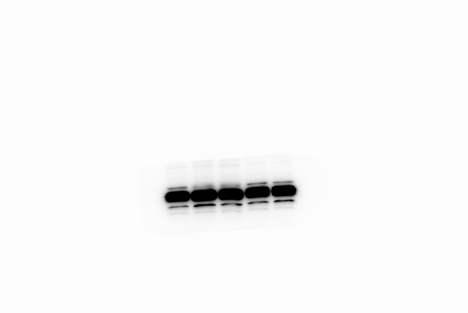


B

**Figure 8**: Cropped target protein bands from full membrane (as shown in Figure 7).

**A**. Target protein band for VCAM1.

**B**. Cropped internal reference protein band (GAPDH).

**Sample 2**

#### 1. Full Membrane Images


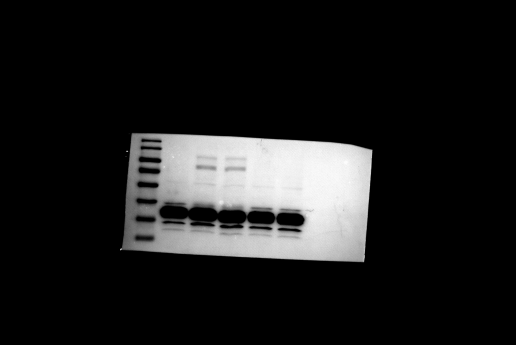


**Figure 9**: Full membrane image of VCAM1 and GAPDH.

#### 2. Cropped Target Protein Bands


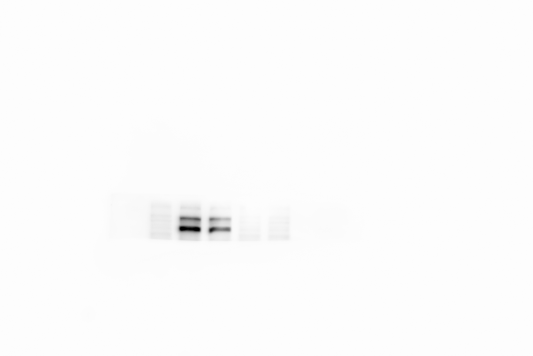


A


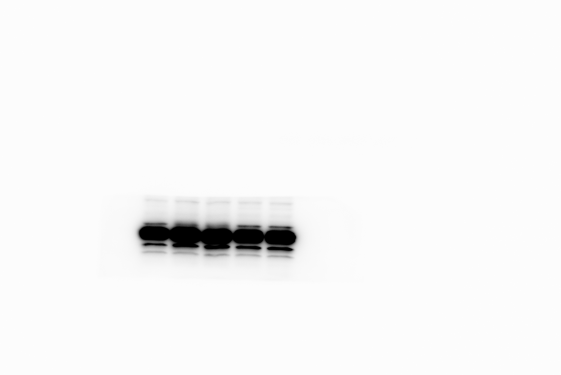


B

**Figure 10**: Cropped target protein bands from full membrane (as shown in Figure 9).

**A**. Target protein band for VCAM1.

**B**. Cropped internal reference protein band (GAPDH).

**Sample 3**

#### 1. Full Membrane Images


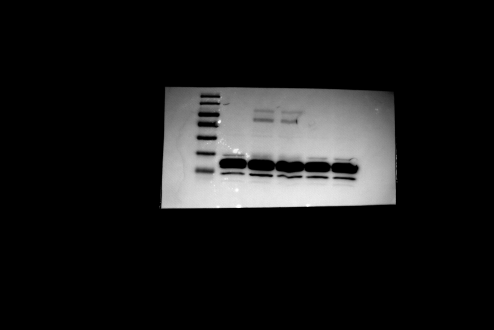


**Figure 11**: Full membrane image of VCAM1 and GAPDH.

#### 2. Cropped Target Protein Bands


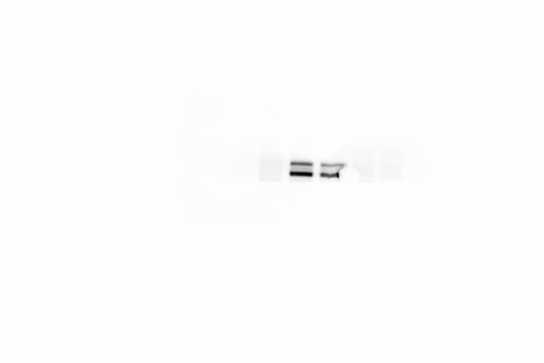


A


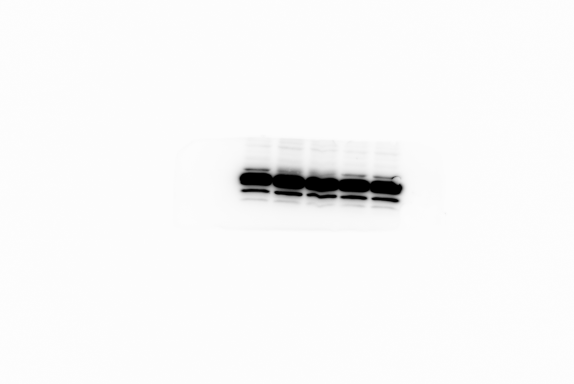


B

**Figure 12**: Cropped target protein bands from full membrane (as shown in Figure 11).

**A**. Target protein band for VCAM1.

**B**. Cropped internal reference protein band (GAPDH).

| **Sample Description** | **Target Protein (Grayscale Value)** | | | | | **Internal Reference (Grayscale Value)** | | | | | **Normalized Value** | | | | |
| --- | --- | --- | --- | --- | --- | --- | --- | --- | --- | --- | --- | --- | --- | --- | --- |
|  | **CON** | **MOD** | **L-dose** | **M-dose** | **H-dose** | **CON** | **MOD** | **L-dose** | **M-dose** | **H-dose** | **CON** | **MOD** | **L-dose** | **M-dose** | **H-dose** |
| Sample 1 | 6440.782 | 53437.836 | 20534.622 | 3104.953 | 3026.196 | 65459.338 | 74739.874 | 74143.045 | 69164.803 | 66515.51 | 0.098393632 | 0.714984293 | 0.276959518 | 0.044892096 | 0.045496096 |
| Sample 2 | 12215.288 | 56734.35 | 29239.744 | 1887.761 | 3630.347 | 65989.894 | 74098.024 | 72220.581 | 67949.359 | 65967.773 | 0.185108465 | 0.765666167 | 0.404867194 | 0.027781881 | 0.055032129 |
| Sample 3 | 8222.146 | 54244.572 | 33131.886 | 4846.489 | 3173.589 | 73744.752 | 74375.459 | 75353.652 | 69726.095 | 69403.066 | 0.111494659 | 0.729334282 | 0.439685206 | 0.069507535 | 0.045726928 |

Table 2: Grayscale values of VCAM1. bands analyzed using ImageJ.

**Western blot bands of MERTK in BV2 cells (Control, Model, and YQHXP high, medium, and low groups)**

**Sample 1**

#### 1. Full Membrane Images


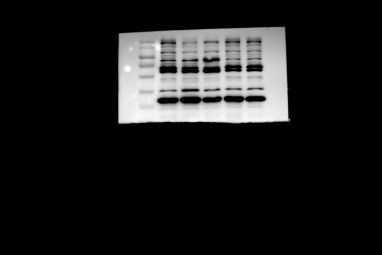


**Figure 13**: Full membrane image of MERTK and GAPDH.

#### 2. Cropped Target Protein Bands


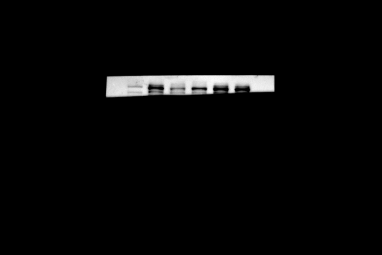


A


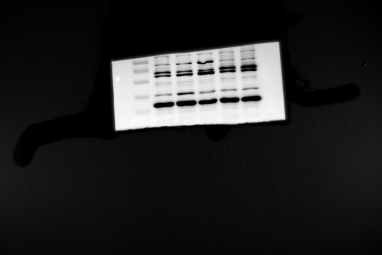


B

**Figure 14**: Cropped target protein bands from full membrane (as shown in Figure 13).

**A**. Target protein band for MERTK.

**B**. Cropped internal reference protein band (GAPDH).

**Sample 2**

#### 1. Full Membrane Images


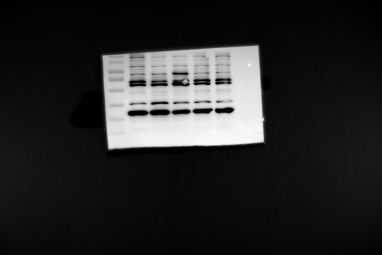


**Figure 15**: Full membrane image of MERTK and GAPDH.

#### 2. Cropped Target Protein Bands


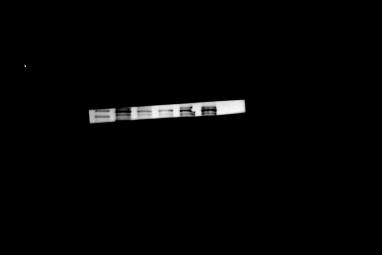


A


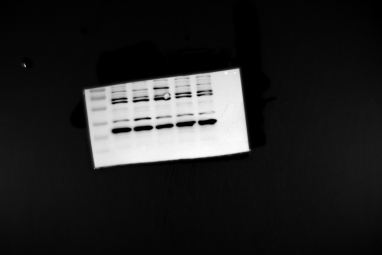


B

**Figure 16**: Cropped target protein bands from full membrane (as shown in Figure 15).

**A**. Target protein band for MERTK.

**B**. Cropped internal reference protein band (GAPDH).

**Sample 3**

#### 1. Full Membrane Images


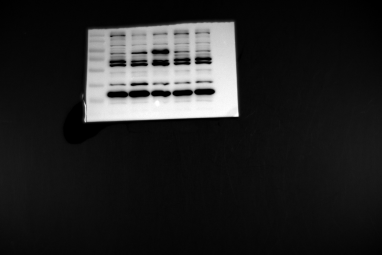


**Figure 17**: Full membrane image of MERTK and GAPDH.

#### 2. Cropped Target Protein Bands


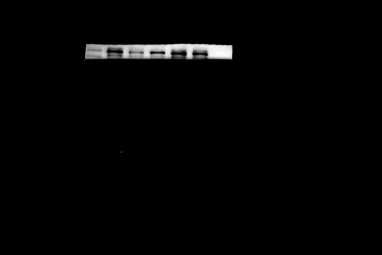


A


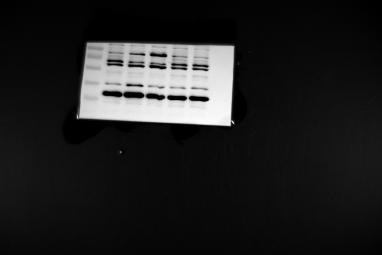


B

**Figure 18**: Cropped target protein bands from full membrane (as shown in Figure 17).

**A**. Target protein band for MERTK.

**B**. Cropped internal reference protein band (GAPDH).

| **Sample Description** | **Target Protein (Grayscale Value)** | | | | | **Internal Reference (Grayscale Value)** | | | | | **Normalized Value** | | | | |
| --- | --- | --- | --- | --- | --- | --- | --- | --- | --- | --- | --- | --- | --- | --- | --- |
|  | **CON** | **MOD** | **L-dose** | **M-dose** | **H-dose** | **CON** | **MOD** | **L-dose** | **M-dose** | **H-dose** | **CON** | **MOD** | **L-dose** | **M-dose** | **H-dose** |
| Sample 1 | 26343.388 | 13538.924 | 16534.217 | 23387.681 | 24181.924 | 31617.095 | 34510.66 | 27954.246 | 33141.61 | 33409.024 | 0.833200773 | 0.392311361 | 0.591474261 | 0.705689343 | 0.723814141 |
| Sample 2 | 28544.539 | 11501.095 | 5556.146 | 16698.439 | 22942.338 | 34980.61 | 31065.782 | 26053.317 | 32079.024 | 28928.731 | 0.816010327 | 0.370217463 | 0.793064099 | 0.520540743 | 0.793064099 |
| Sample 3 | 29338.095 | 14033.51 | 14724.853 | 22249.267 | 19020.317 | 32348.317 | 36456.882 | 30511.539 | 26427.439 | 25691.974 | 0.906943474 | 0.384934455 | 0.740321355 | 0.841900231 | 0.740321355 |

Table 3: Grayscale values of MERTK. bands analyzed using ImageJ.

**Western blot bands of C1QB in hippocampal tissue from Control, Model, and YQHXP groups of mice.**

**Sample 1**

#### 1. Full Membrane Images


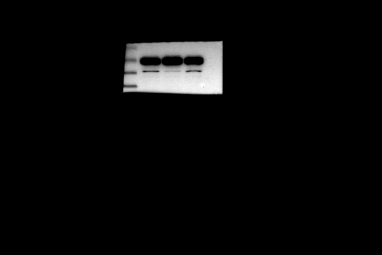


**Figure 19**: Full membrane image of C1QB and GAPDH.

#### 2. Cropped Target Protein Bands


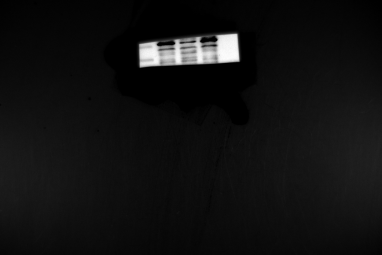


A


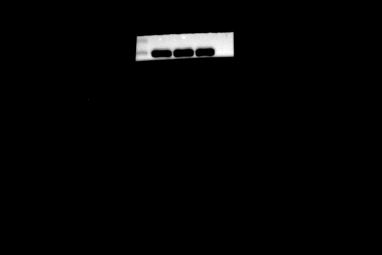


B

**Figure 20**: Cropped target protein bands from full membrane (as shown in Figure 19).

**A**. Target protein band for C1QB.

**B**. Cropped internal reference protein band (GAPDH).

**Sample 2**

#### 1. Full Membrane Images


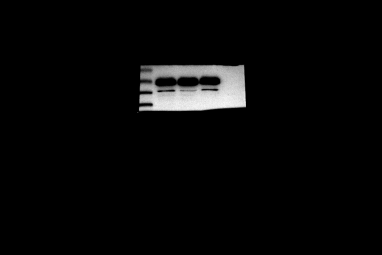


**Figure 21**: Full membrane image of C1QB and GAPDH.

#### 2. Cropped Target Protein Bands


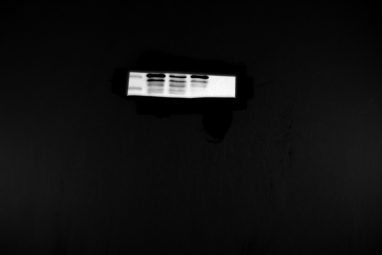


A


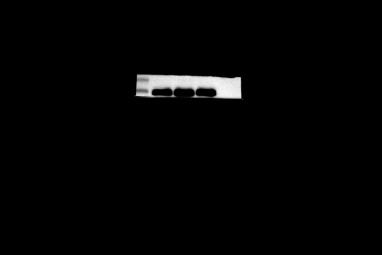


B

**Figure 22**: Cropped target protein bands from full membrane (as shown in Figure 21).

**A**. Target protein band for C1QB.

**B**. Cropped internal reference protein band (GAPDH).

**Sample 3**

#### 1. Full Membrane Images


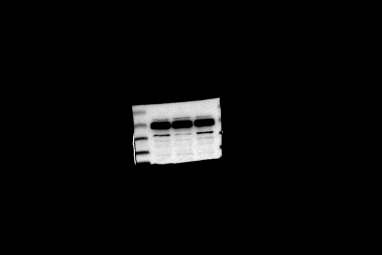


**Figure 23**: Full membrane image of C1QB and GAPDH.

#### 2. Cropped Target Protein Bands


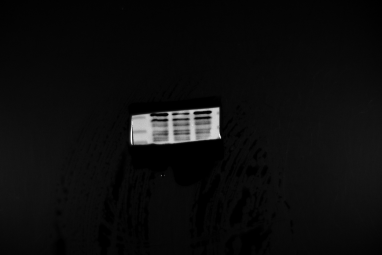


A


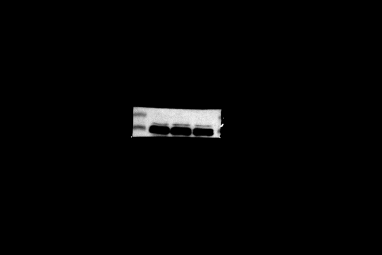


B

**Figure 24**: Cropped target protein bands from full membrane (as shown in Figure 23).

**A**. Target protein band for C1QB.

**B**. Cropped internal reference protein band (GAPDH).

| **Sample Description** | **Target Protein (Grayscale Value)** | | | **Internal Reference (Grayscale Value)** | | | **Normalized Value** | | |
| --- | --- | --- | --- | --- | --- | --- | --- | --- | --- |
|  | **CON** | **MOD** | **YQHXP** | **CON** | **MOD** | **YQHXP** | **CON** | **MOD** | **YQHXP** |
| Sample 1 | 48499.765 | 24571.258 | 48572.693 | 48863.551 | 54395.744 | 54575.38 | 0.992555064 | 0.451712877 | 0.890011082 |
| Sample 2 | 54474.765 | 31607.258 | 43995.401 | 53178.936 | 57767.966 | 52208.108 | 1.024367336 | 0.547141611 | 0.842692882 |
| Sample 3 | 56835.409 | 28885.836 | 44256.087 | 59806.844 | 61702.217 | 55934.359 | 0.950316138 | 0.468149078 | 0.791214699 |

Table 4: Grayscale values of C1QB. bands analyzed using ImageJ.

**Western blot bands of VCAM1 in hippocampal tissue from Control, Model, and YQHXP groups of mice.**

**Sample 1**

#### 1. Full Membrane Images


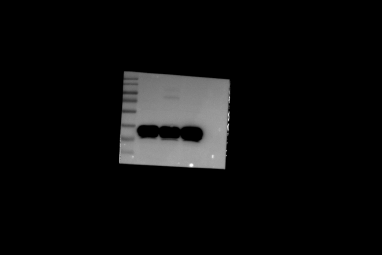


**Figure 25**: Full membrane image of VCAM1 and GAPDH.

#### 2. Cropped Target Protein Bands


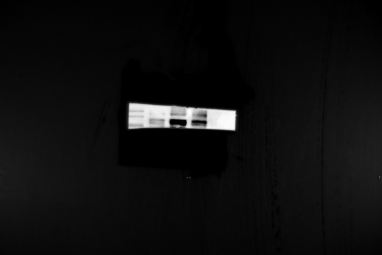


A


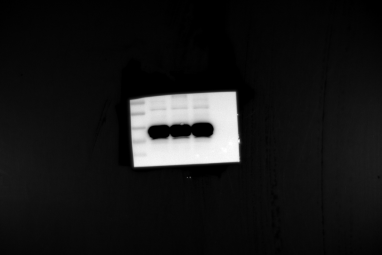


B

**Figure 26**: Cropped target protein bands from full membrane (as shown in Figure 25).

**A**. Target protein band for VCAM1.

**B**. Cropped internal reference protein band (GAPDH).

**Sample 2**

#### 1. Full Membrane Images


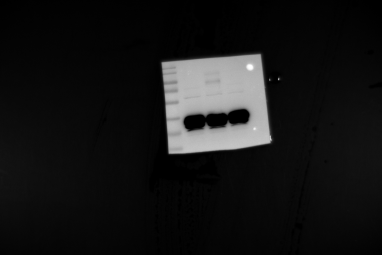


**Figure 27**: Full membrane image of VCAM1 and GAPDH.

#### 2. Cropped Target Protein Bands


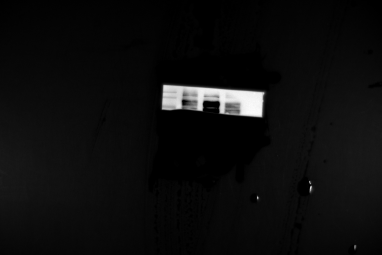


A


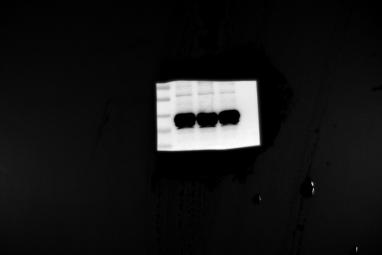


B

**Figure 28**: Cropped target protein bands from full membrane (as shown in Figure 27).

**A**. Target protein band for VCAM1.

**B**. Cropped internal reference protein band (GAPDH).

**Sample 3**

#### 1. Full Membrane Images


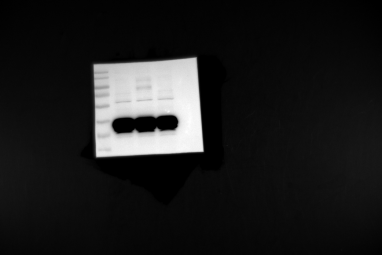


**Figure 29**: Full membrane image of VCAM1 and GAPDH.

#### 2. Cropped Target Protein Bands


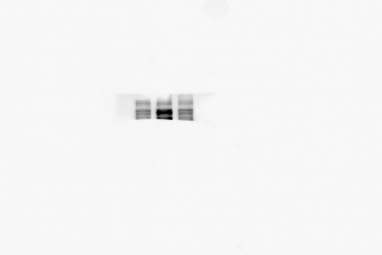


A


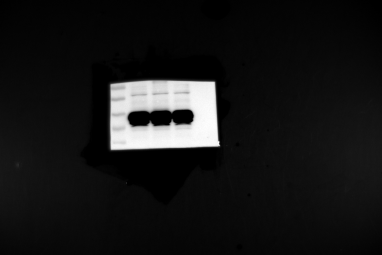


B

**Figure 30**: Cropped target protein bands from full membrane (as shown in Figure 29).

**A**. Target protein band for VCAM1.

**B**. Cropped internal reference protein band (GAPDH).

| **Sample Description** | **Target Protein (Grayscale Value)** | | | **Internal Reference (Grayscale Value)** | | | **Normalized Value** | | |
| --- | --- | --- | --- | --- | --- | --- | --- | --- | --- |
|  | **CON** | **MOD** | **YQHXP** | **CON** | **MOD** | **YQHXP** | **CON** | **MOD** | **YQHXP** |
| Sample 1 | 15470.823 | 50584.693 | 37042.359 | 53688.439 | 53100.853 | 56292.652 | 0.2881593 | 0.95261545 | 0.658031869 |
| Sample 2 | 33620.794 | 48672.116 | 23911.288 | 55656.681 | 55443.338 | 54255.48 | 0.60407472 | 0.877871314 | 0.440716551 |
| Sample 3 | 24645.966 | 48538.643 | 26265.823 | 62027.681 | 61933.711 | 58855.803 | 0.397338182 | 0.783719274 | 0.446274142 |

Table 5: Grayscale values of VCAM1. bands analyzed using ImageJ.

**Western blot bands of MERTK in hippocampal tissue from Control, Model, and YQHXP groups of mice.**

**Sample 1**

#### 1. Full Membrane Images


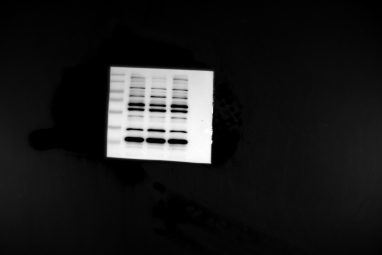


**Figure 31**: Full membrane image of MERTK and GAPDH.

#### 2. Cropped Target Protein Bands


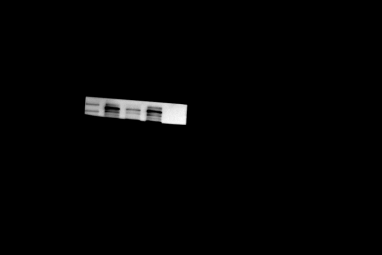


A


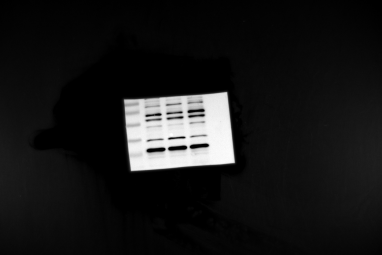


B

**Figure 32**: Cropped target protein bands from full membrane (as shown in Figure 31).

**A**. Target protein band for MERTK.

**B**. Cropped internal reference protein band (GAPDH).

**Sample 2**

#### 1. Full Membrane Images


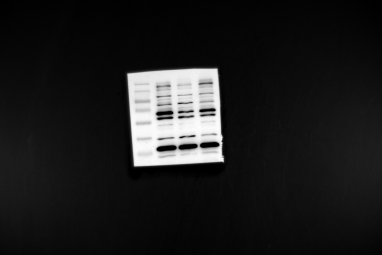


**Figure 33**: Full membrane image of MERTK and GAPDH.

#### 2. Cropped Target Protein Bands


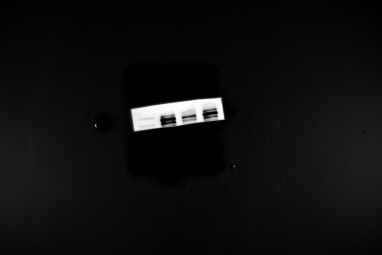


A


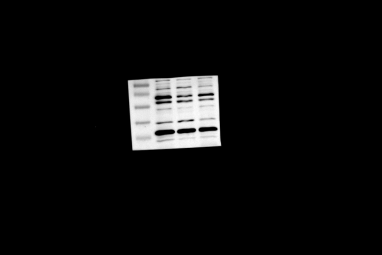


B

**Figure 34**: Cropped target protein bands from full membrane (as shown in Figure 33).

**A**. Target protein band for MERTK.

**B**. Cropped internal reference protein band (GAPDH).

**Sample 3**

#### 1. Full Membrane Images


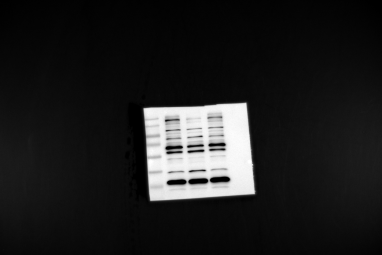


**Figure 35**: Full membrane image of MERTK and GAPDH.

#### 2. Cropped Target Protein Bands


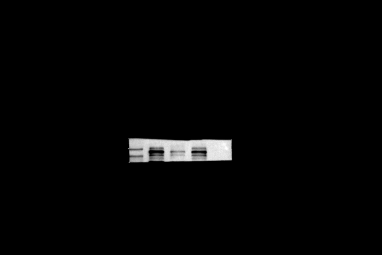


A


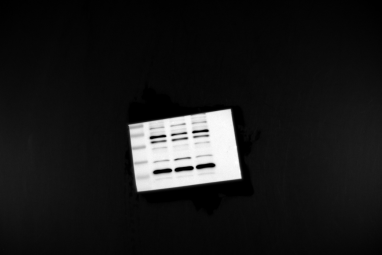


B

**Figure 36**: Cropped target protein bands from full membrane (as shown in Figure 35).

**A**. Target protein band for MERTK.

**B**. Cropped internal reference protein band (GAPDH).

| **Sample Description** | **Target Protein (Grayscale Value)** | | | **Internal Reference (Grayscale Value)** | | | **Normalized Value** | | |
| --- | --- | --- | --- | --- | --- | --- | --- | --- | --- |
|  | **CON** | **MOD** | **YQHXP** | **CON** | **MOD** | **YQHXP** | **CON** | **MOD** | **YQHXP** |
| Sample 1 | 48144.016 | 17308.723 | 41280.401 | 54392.53 | 55494.48 | 53523.915 | 0.885121836 | 0.311899904 | 0.771251524 |
| Sample 2 | 43741.894 | 23304.915 | 38549.409 | 56719.309 | 46520.53 | 41354.459 | 0.771199346 | 0.500959791 | 0.932170555 |
| Sample 3 | 46279.229 | 15204.35 | 36798.844 | 54333.723 | 52645.602 | 55313.602 | 0.851758842 | 0.288805701 | 0.665276581 |

Table 6: Grayscale values of MERTK. bands analyzed using ImageJ.

**Western blot bands of C1QB in BV2 cells (Control, Model, YQHXP, UNC2250, UNC2250+YQHXP, Rutin, and Rutin+YQHXP groups).**

**Sample 1**

#### 1. Full Membrane Images


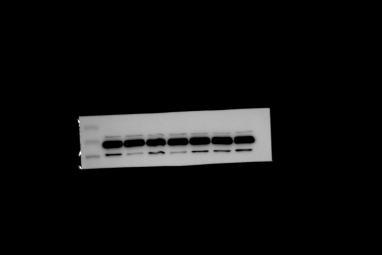


**Figure 37**: Full membrane image of C1QB and GAPDH.

#### 2. Cropped Target Protein Bands


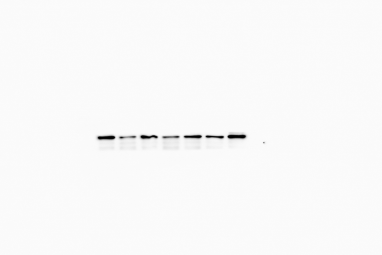


A


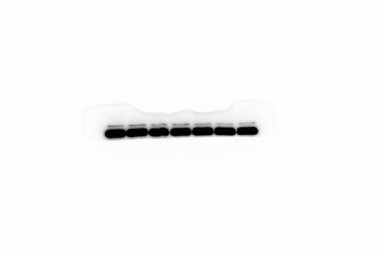


B

**Figure 38**: Cropped target protein bands from full membrane (as shown in Figure 37).

**A**. Target protein band for C1QB.

**B**. Cropped internal reference protein band (GAPDH).

**Sample 2**

#### 1. Full Membrane Images


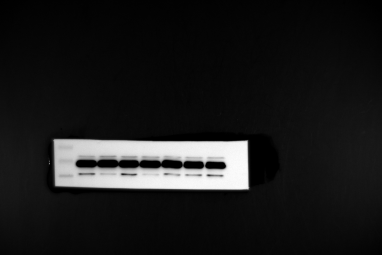


**Figure 39**: Full membrane image of C1QB and GAPDH.

#### 2. Cropped Target Protein Bands


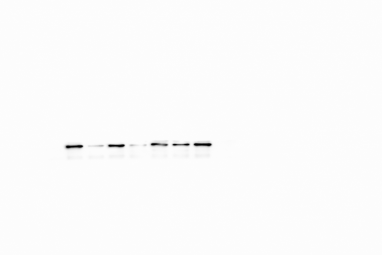


A


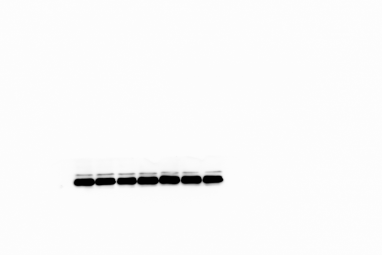


B

**Figure 40**: Cropped target protein bands from full membrane (as shown in Figure 39).

**A**. Target protein band for C1QB.

**B**. Cropped internal reference protein band (GAPDH).

**Sample 3**

#### 1. Full Membrane Images


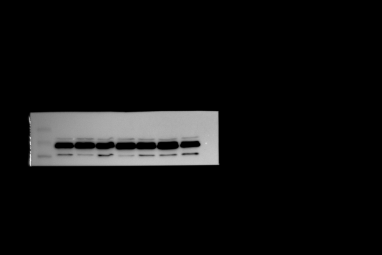


**Figure 41**: Full membrane image of C1QB and GAPDH.

#### 2. Cropped Target Protein Bands


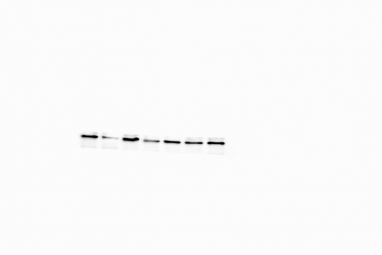


A


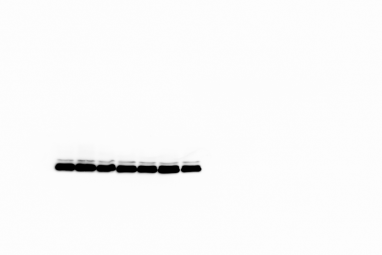


B

**Figure 42**: Cropped target protein bands from full membrane (as shown in Figure 41).

**A**. Target protein band for C1QB.

**B**. Cropped internal reference protein band (GAPDH).

| **Sample Description** | **Target Protein (Grayscale Value)** | | | | |  |  | **Internal Reference (Grayscale Value)** | | | | |  |  | **Normalized Value** | | | | | | |
| --- | --- | --- | --- | --- | --- | --- | --- | --- | --- | --- | --- | --- | --- | --- | --- | --- | --- | --- | --- | --- | --- |
|  | **CON** | **MOD** | **YQHXP** | **TNFα** | **TNFα+YQHXP** | **Rutin** | **Rutin+YQHXP** | **CON** | **MOD** | **YQHXP** | **TNFα** | **TNFα+YQHXP** | **Rutin** | **Rutin+YQHXP** | **CON** | **MOD** | **YQHXP** | **TNFα** | **TNFα+YQHXP** | **Rutin** | **Rutin+YQHXP** |
| Sample 1 | 18944.175 | 7812.69 | 15512.175 | 8976.69 | 15817.761 | 12578.811 | 22087.175 | 25371.225 | 26830.74 | 23288.255 | 26757.154 | 24015.497 | 25413.79 | 24388.74 | 0.746679555 | 0.291184291 | 0.666094347 | 0.335487474 | 0.65864808 | 0.494960059 | 0.905630016 |
| Sample 2 | 19328.761 | 1935.669 | 15909.054 | 1845.447 | 14158.589 | 11686.64 | 20276.175 | 24551.811 | 22552.962 | 21754.861 | 26543.619 | 26782.255 | 25456.497 | 24060.397 | 0.787264166 | 0.085827706 | 0.73128732 | 0.069525071 | 0.528655597 | 0.459082803 | 0.842719885 |
| Sample 3 | 17602.589 | 5336.397 | 20248.004 | 8944.225 | 14706.175 | 14550.175 | 17563.933 | 24783.912 | 29396.882 | 21412.69 | 26895.589 | 23569.196 | 27074.933 | 21284.569 | 0.710242556 | 0.181529354 | 0.945607675 | 0.332553602 | 0.623957432 | 0.537403915 | 0.825195615 |

Table 7: Grayscale values of C1QB. bands analyzed using ImageJ.

**Western blot bands of VCAM1 in BV2 cells (Control, Model, YQHXP, UNC2250, UNC2250+YQHXP, Rutin, and Rutin+YQHXP groups).**

**Sample 1**

#### 1. Full Membrane Images


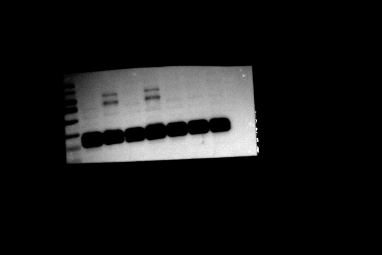


**Figure 43**: Full membrane image of VCAM1 and GAPDH.

#### 2. Cropped Target Protein Bands


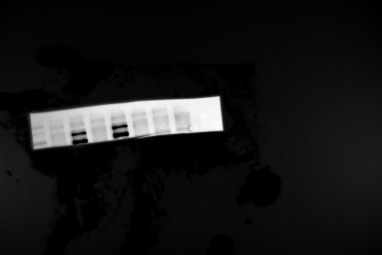


A


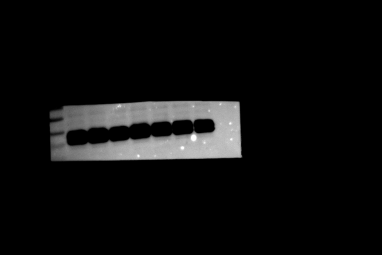


B

**Figure 44**: Cropped target protein bands from full membrane (as shown in Figure 43).

**A**. Target protein band for VCAM1.

**B**. Cropped internal reference protein band (GAPDH).

**Sample 2**

#### 1. Full Membrane Images


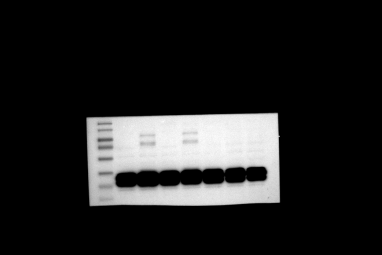


**Figure 45**: Full membrane image of VCAM1 and GAPDH.

#### 2. Cropped Target Protein Bands


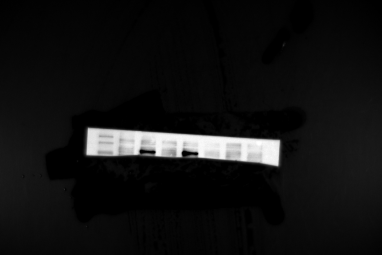


A


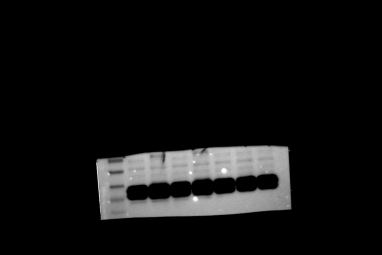


B

**Figure 46**: Cropped target protein bands from full membrane (as shown in Figure 45).

**A**. Target protein band for VCAM1.

**B**. Cropped internal reference protein band (GAPDH).

**Sample 3**

#### 1. Full Membrane Images


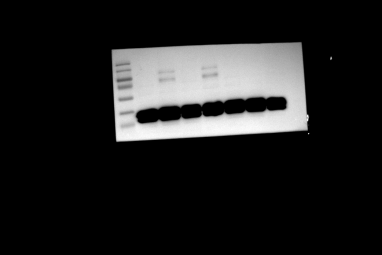


**Figure 47**: Full membrane image of VCAM1 and GAPDH.

#### 2. Cropped Target Protein Bands


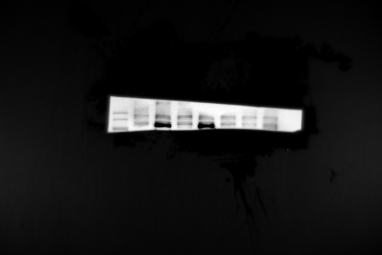


A


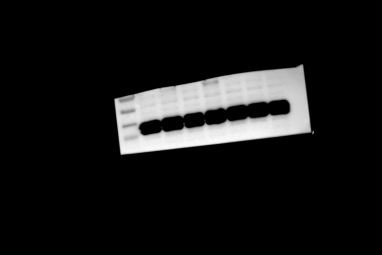


B

**Figure 48**: Cropped target protein bands from full membrane (as shown in Figure 47).

**A**. Target protein band for VCAM1.

**B**. Cropped internal reference protein band (GAPDH).

| **Sample Description** | **Target Protein (Grayscale Value)** | | | | |  |  | **Internal Reference (Grayscale Value)** | | | | |  |  | **Normalized Value** | | | | | | |
| --- | --- | --- | --- | --- | --- | --- | --- | --- | --- | --- | --- | --- | --- | --- | --- | --- | --- | --- | --- | --- | --- |
|  | **CON** | **MOD** | **YQHXP** | **TNFα** | **TNFα+YQHXP** | **Rutin** | **Rutin+YQHXP** | **CON** | **MOD** | **YQHXP** | **TNFα** | **TNFα+YQHXP** | **Rutin** | **Rutin+YQHXP** | **CON** | **MOD** | **YQHXP** | **TNFα** | **TNFα+YQHXP** | **Rutin** | **Rutin+YQHXP** |
| Sample 1 | 9432.255 | 20553.832 | 8393.104 | 20874.368 | 6498.518 | 5211.104 | 6122.104 | 29875.276 | 25162.711 | 27956.69 | 23049.225 | 27989.004 | 25552.447 | 22697.205 | 0.3157211 | 0.816836946 | 0.300218087 | 0.905642945 | 0.232181109 | 0.203937572 | 0.269729423 |
| Sample 2 | 2599.447 | 19282.882 | 2128.619 | 13599.589 | 2087.79 | 4699.518 | 1544.841 | 24447.619 | 27154.497 | 25765.569 | 24657.518 | 25170.69 | 22153.468 | 25135.024 | 0.106327205 | 0.710117444 | 0.082614865 | 0.551539251 | 0.082945283 | 0.212134642 | 0.061461688 |
| Sample 3 | 5881.104 | 20563.125 | 7501.518 | 19677.418 | 6085.811 | 3709.518 | 7908.983 | 23260.154 | 25846.154 | 24521.841 | 26869.74 | 26952.326 | 27431.376 | 26397.447 | 0.252840286 | 0.795597093 | 0.305911697 | 0.732326327 | 0.225799102 | 0.135229017 | 0.299611663 |

Table 8: Grayscale values of VCAM1. bands analyzed using ImageJ.

**Western blot bands of MERTK in BV2 cells (Control, Model, YQHXP, UNC2250, UNC2250+YQHXP, Rutin, and Rutin+YQHXP groups).**

**Sample 1**

#### 1. Full Membrane Images


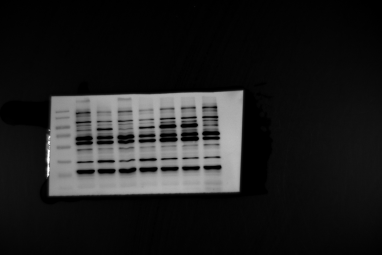


**Figure 49**: Full membrane image of MERTK and GAPDH.

#### 2. Cropped Target Protein Bands


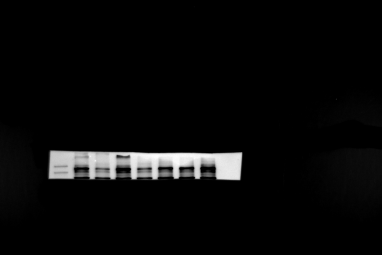


A


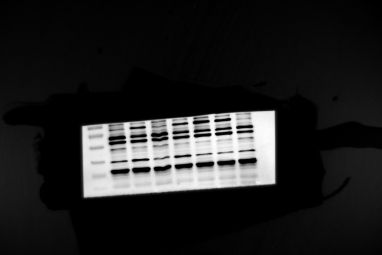


B

**Figure 50**: Cropped target protein bands from full membrane (as shown in Figure 49).

**A**. Target protein band for MERTK.

**B**. Cropped internal reference protein band (GAPDH).

**Sample 2**

#### 1. Full Membrane Images


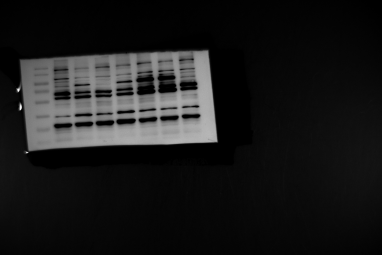


**Figure 51**: Full membrane image of MERTK and GAPDH.

#### 2. Cropped Target Protein Bands


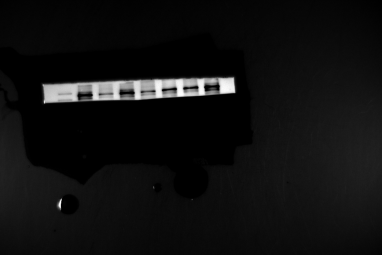


A


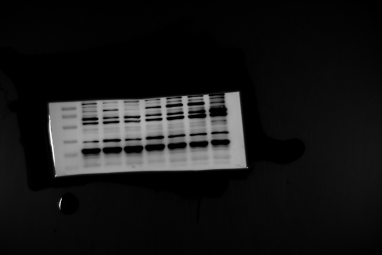


B

**Figure 52**: Cropped target protein bands from full membrane (as shown in Figure 51).

**A**. Target protein band for MERTK.

**B**. Cropped internal reference protein band (GAPDH).

**Sample 3**

#### 1. Full Membrane Images


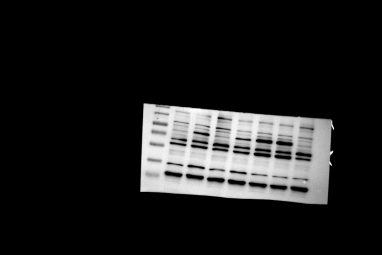


**Figure 53** Full membrane image of MERTK and GAPDH.

#### 2. Cropped Target Protein Bands


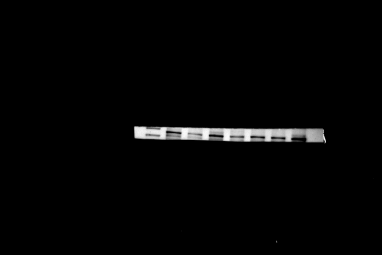


A


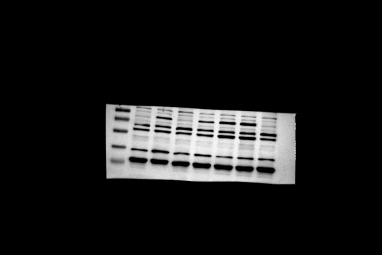


B

**Figure 54**: Cropped target protein bands from full membrane (as shown in Figure 53).

**A**. Target protein band for MERTK.

**B**. Cropped internal reference protein band (GAPDH).

| **Sample Description** | **Target Protein (Grayscale Value)** | | | | |  |  | **Internal Reference (Grayscale Value)** | | | | |  |  | **Normalized Value** | | | | | | |
| --- | --- | --- | --- | --- | --- | --- | --- | --- | --- | --- | --- | --- | --- | --- | --- | --- | --- | --- | --- | --- | --- |
|  | **CON** | **MOD** | **YQHXP** | **TNFα** | **TNFα+YQHXP** | **Rutin** | **Rutin+YQHXP** | **CON** | **MOD** | **YQHXP** | **TNFα** | **TNFα+YQHXP** | **Rutin** | **Rutin+YQHXP** | **CON** | **MOD** | **YQHXP** | **TNFα** | **TNFα+YQHXP** | **Rutin** | **Rutin+YQHXP** |
| Sample 1 | 17971.933 | 9616.811 | 20359.024 | 10406.225 | 16007.66 | 15770.761 | 21328.811 | 22226.933 | 22425.64 | 19452.225 | 19469.64 | 21046.225 | 20325.518 | 23323.933 | 0.808565581 | 0.428831061 | 1.046616724 | 0.534484716 | 0.760595309 | 0.775909426 | 0.91446031 |
| Sample 2 | 20293.104 | 10338.811 | 19483.024 | 11072.832 | 12742.004 | 12492.811 | 18573.175 | 22019.69 | 22863.276 | 24889.761 | 24425.296 | 20969.175 | 21330.175 | 20852.175 | 0.921588996 | 0.452201644 | 0.782772643 | 0.453334609 | 0.607654045 | 0.585687225 | 0.890706845 |
| Sample 3 | 19083.054 | 10470.246 | 16129.418 | 9150.983 | 13002.246 | 14375.731 | 21121.196 | 21097.761 | 21869.418 | 18784.104 | 18977.711 | 19859.761 | 21173.761 | 24126.175 | 0.904506123 | 0.478761986 | 0.858673802 | 0.482196351 | 0.654703045 | 0.678940836 | 0.875447351 |

Table 9: Grayscale values of MERTK. bands analyzed using ImageJ.
