# Supplementary material for: YiQi-HuoXue prescription ameliorates LPS-induced sepsis-associated encephalopathy via VCAM-1–mediated microglial efferocytosis
Source: Front Immunol. 2026 Apr 1;17:1792688. doi: 10.3389/fimmu.2026.1792688 (PMC13079163; doi:10.3389/fimmu.2026.1792688)
Supplement: Supplementary file 2 [file DataSheet1.docx]

**Supplementary Materials**

**
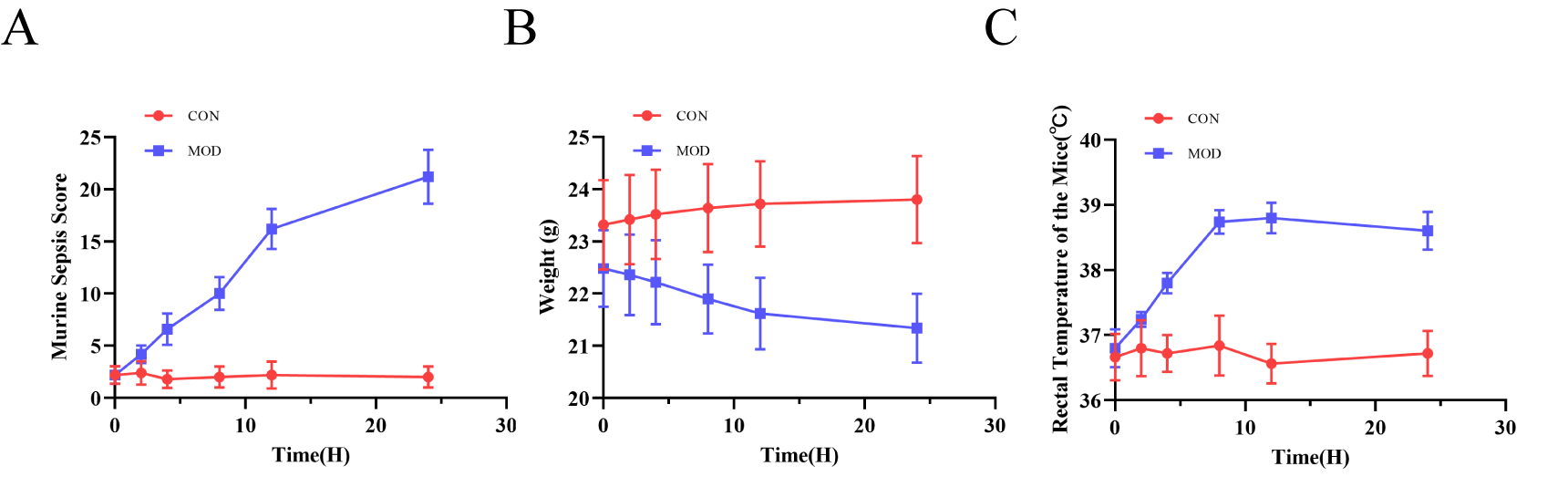
**

### *Figure S1. Quality control of the LPS-induced SAE model prior to intervention.*

*(A) Time-course changes in Murine Sepsis Score (MSS) after intraperitoneal injection of LPS (Model, MOD) or sterile saline (Control, CON).
(B) Time-course changes in body weight.
(C) Time-course changes in rectal temperature.*

*Table 1 Constituent identification of YQHXP*

| **NO.** | **Name of Prototype** | **Retention time (min)** | **Adducts** | **Experimental m/z** | **Theoretical m/z** | **ppm** |
| --- | --- | --- | --- | --- | --- | --- |
|  |  |  |  |  |  |  |
| 1 | Gallic acid | 2.86 | [M-H]- | 169.0154 | 169.0142 | 1.3 |
| 2 | Neochlorogenic acid | 5.81 | [M-H]- | 353.0883 | 353.0878 | 1.4 |
| 3 | Chlorogenic acid | 7.23 | [M-H]- | 353.0876 | 353.0878 | -0.6 |
| 4 | Cryptochlorogenic acid | 7.53 | [M-H]- | 353.0875 | 353.0878 | -0.9 |
| 5 | Vanillic acid | 7.72 | [M-H]- | 167.0358 | 167.035 | 4.9 |
| 6 | Caffeic acid | 7.96 | [M-H]- | 179.0359 | 179.035 | 5.1 |
| 7 | 1,3-di-O-Dicaffeoylquinic acid | 8.77 | [M-H]- | 515.1197 | 515.1195 | 0.4 |
| 8 | Quinquenoside F1 | 9.48 | [M+FA-H]- | 863.5012 | 863.501 | 0.3 |
| 9 | Aloe-emodin-8-glucopyranoside | 10.15 | [M-H]- | 431.0978 | 431.0984 | -1.3 |
| 10 | Ferulic acid | 10.38 | [M-H]- | 193.0506 | 193.0506 | -0.2 |
| 11 | Rhein-8-O-β-D-glucopyranoside | 10.45 | [M-H]- | 445.0784 | 445.0776 | 1.7 |
| 12 | / | 11.22 | [M+H]+ | 263.0589 | 263.0584 | 2 |
| 13 | Notoginsenoside R1 | 12.27 | [M+FA-H]- | 977.5365 | 977.5321 | 4.5 |
| 14 | Chuanxiongoside B | 12.39 | [M+FA-H]- | 549.219 | 549.2189 | 0.2 |
| 15 | Senkyunolide I | 12.45 | [M-H2O+H]+ | 207.1016 | 207.1016 | 0 |
| 16 | Ginsenoside Rg1 | 12.76 | [M+FA-H]- | 845.4933 | 845.4904 | 3.4 |
| 17 | Ginsenoside Re | 12.79 | [M+FA-H]- | 991.5493 | 991.5483 | 1 |
| 18 | Senkyunolide H | 12.99 | [M-H2O+H]+ | 207.1017 | 207.1016 | 0.6 |
| 19 | Notoginsenoside A | 13.23 | [M+FA-H]- | 1169.593 | 1169.5961 | -2.6 |
| 20 | Chrysophanol-1-O-glucoside | 14.35 | [M-H]- | 415.1035 | 415.1035 | 0.1 |
| 21 | Emodin-8-glucoside | 14.4 | [M-H]- | 431.1008 | 431.0984 | 5.6 |
| 22 | Emodin 1-O-β-D-glucopyranoside | 14.55 | [M-H]- | 431.0984 | 431.0984 | 0.1 |
| 23 | Chrysophanol 8-O-β-D-glucoside | 14.68 | [M-H]- | 415.1048 | 415.1035 | 3.2 |
| 24 | Ginsenoside Ra3 | 15.71 | [M+2FA-2H]2- | 665.3242 | 665.3202 | 6 |
| 25 | Ginsenoside Rf | 15.82 | [M+FA-H]- | 845.4911 | 845.4904 | 0.8 |
| 26 | 3-[5′-(2-Carboxyethenyl)-2′,6-dihydroxy-3′,5-dimethoxy[1,1′-biphenyl]-3-yl]-2-[4-(2-carboxyethenyl)-2-methoxyphenoxy]-2-propenoic acid | 15.91 | [M-H]- | 577.1374 | 577.1352 | 3.8 |
| 27 | Notoginsenoside Fa | 16.15 | [M+2FA-2H]2- | 665.322 | 665.3202 | 2.7 |
| 28 | Ginsenoside F5 | 16.38 | [M+FA-H]- | 815.4824 | 815.4798 | 3.1 |
| 29 | Ginsenoside Rb1 | 16.75 | [M+FA-H]- | 1153.6082 | 1153.6011 | 6.1 |
| 30 | Ginsenoside Rg2 | 16.92 | [M+FA-H]- | 829.4954 | 829.4955 | -0.1 |
| 31 | Ginsenoside F1 | 17.08 | [M+FA-H]- | 683.4376 | 683.4376 | 0 |
| 32 | Ginsenoside Rc | 17.18 | [M+FA-H]- | 1123.5973 | 1123.5906 | 6 |
| 33 | Ginsenoside Ro | 17.42 | [M-H]- | 955.4886 | 955.4908 | -2.3 |
| 34 | Ginsenoside Rh1 | 17.47 | [M+FA-H]- | 683.4405 | 683.4376 | 4.3 |
| 35 | Ginsenoside Rb2 | 17.63 | [M+FA-H]- | 1123.5964 | 1123.5906 | 5.2 |
| 36 | Ginsenoside Rb3 | 17.76 | [M+FA-H]- | 1123.6001 | 1123.5906 | 8.5 |
| 37 | Quinquenoside R1 | 18.16 | [M+FA-H]- | 1195.6126 | 1195.6117 | 0.7 |
| 38 | Pseudoginsenoside Rh1 | 18.42 | [M+FA-H]- | 683.4373 | 683.4376 | -0.4 |
| 39 | Ginsenoside Rd | 18.68 | [M+FA-H]- | 991.5515 | 991.5483 | 3.2 |
| 40 | Ginsenoside Rs1 | 19.12 | [M+FA-H]- | 1165.6045 | 1165.6011 | 2.9 |
| 41 | Gypenoside XVII | 19.58 | [M+FA-H]- | 991.5506 | 991.5483 | 2.3 |
| 42 | Rhein | 20.24 | [M-H]- | 283.0255 | 283.0248 | 2.4 |
| 43 | Notoginsenoside T5 | 20.98 | [M+FA-H]- | 797.469 | 797.4693 | -0.4 |
| 44 | Ginsenoside Rg6 | 21.3 | [M+FA-H]- | 811.4875 | 811.4849 | 3.2 |
| 45 | Hebevinoside VI | 21.39 | [M+FA-H]- | 797.4707 | 797.4693 | 1.8 |
| 46 | Ginsenoside Rg4 | 21.68 | [M+FA-H]- | 811.4836 | 811.4849 | -1.6 |
| 47 | Ginsenoside Rk3 | 22.05 | [M+FA-H]- | 665.4282 | 665.427 | 1.8 |
| 48 | Senkyunolide A | 22.36 | [M+H]+ | 193.1225 | 193.1223 | 1 |
| 49 | Ginsenoside Rh4 | 22.53 | [M+FA-H]- | 665.4292 | 665.427 | 3.3 |
| 50 | Zingibroside R1 | 22.81 | [M-H]- | 793.4388 | 793.438 | 1 |
| 51 | 20(S)-Ginsenoside Rg3 | 23.69 | [M+FA-H]- | 829.4951 | 829.4955 | -0.5 |
| 52 | 20(R)-Ginsenoside Rg3 | 24.01 | [M+FA-H]- | 829.4968 | 829.4955 | 1.6 |
| 53 | Ligustilide | 25.37 | [M+H]+ | 191.1061 | 191.1067 | -2.9 |
| 54 | Gingerglycolipid B | 26.9 | [M+FA-H]- | 723.3818 | 723.3809 | 1.3 |
| 55 | 1-Linoleoyl-sn-glycero-3-phosphoethanolamine | 27.21 | [M-H]- | 476.2792 | 476.2783 | 2 |
| 56 | 1-Linoleoyl-sn-glycero-3-phosphocholine | 27.37 | [M+FA-H]- | 564.3335 | 564.3307 | 5 |
| 57 | Ginsenoside Rg5 | 27.55 | [M+FA-H]- | 811.4835 | 811.4849 | -1.8 |
| 58 | Ginsenoside Rk1 | 27.97 | [M+FA-H]- | 811.4878 | 811.4849 | 3.5 |


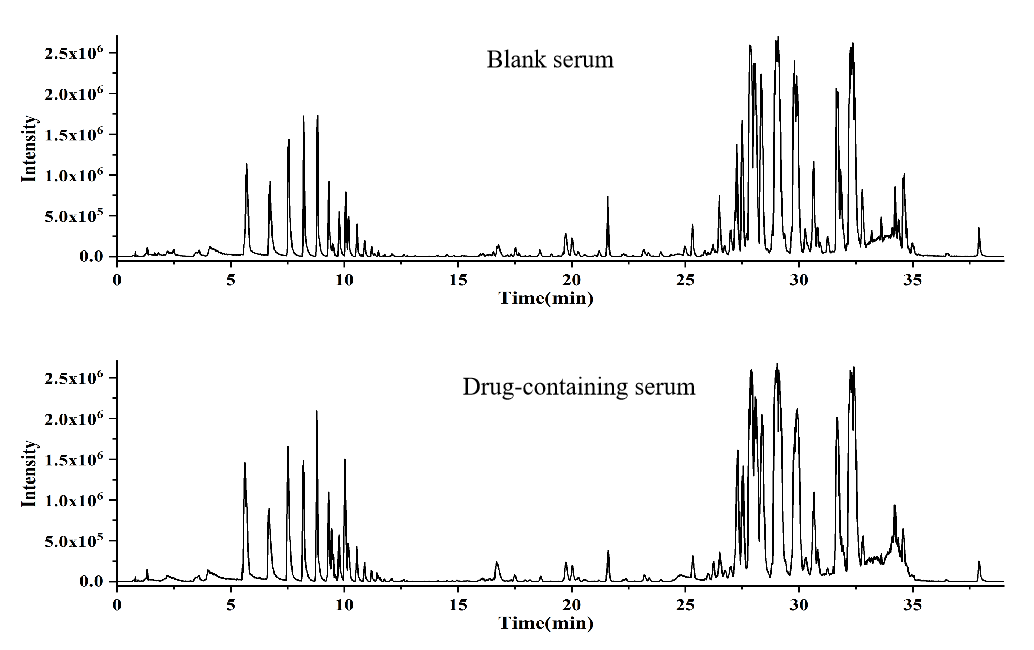


*Fig. S2 Base peak chromatogram (BPC) of YQHXP sample obtained by UPLC-HRMS in positive-ion mode.*


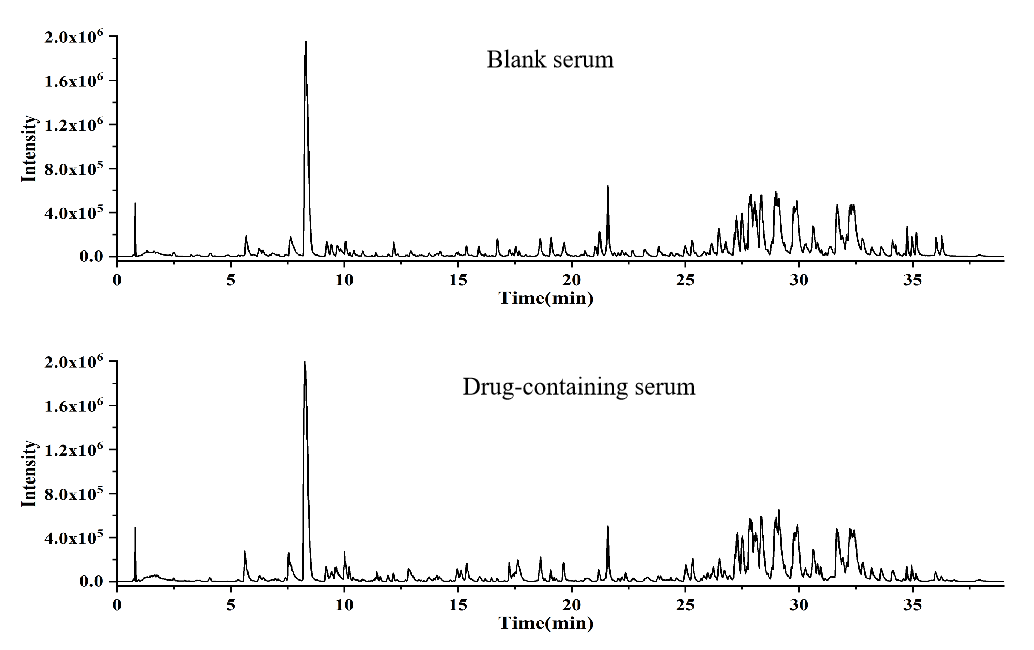


*Fig. S3 Base peak chromatogram (BPC) of YQHXP obtained by UPLC-HRMS in negative-ion mode.*


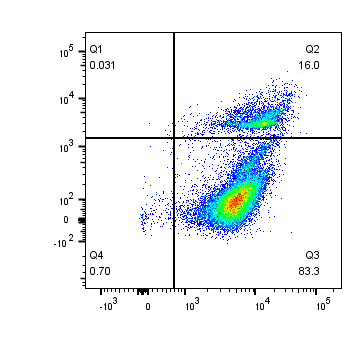


*Fig. S4. Apoptosis assay of HT22 cells performed prior to BV2–HT22 co-culture to ensure apoptotic status of HT22 cells.*


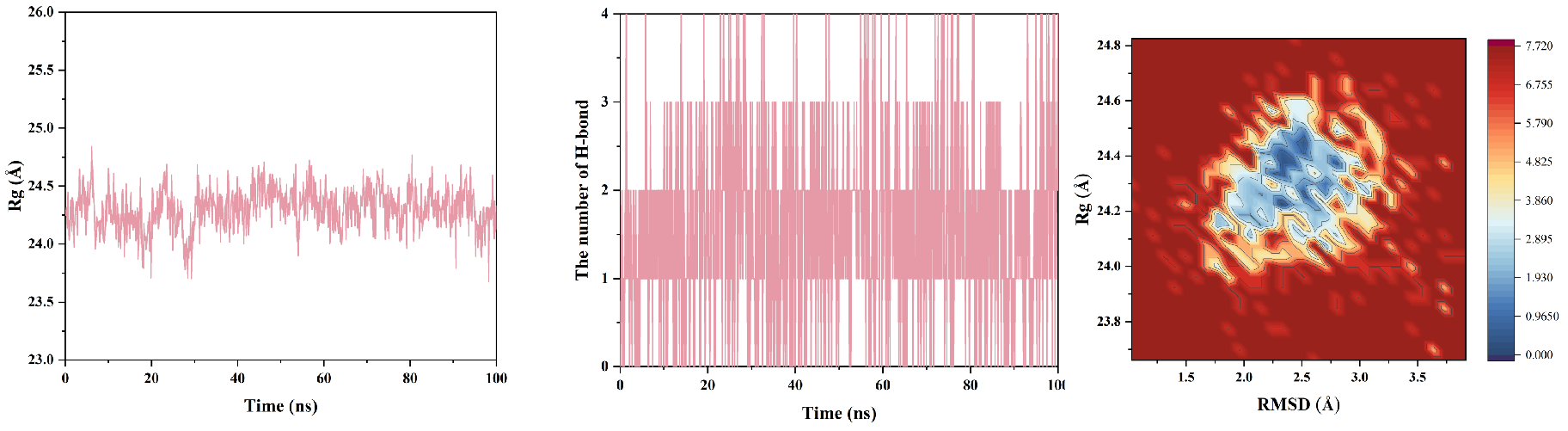


*Fig. S5. Molecular dynamics simulation of the ferulic acid–VCAM1 complex: radius of gyration (Rg), hydrogen bond number curve, and 2D free energy landscape.*


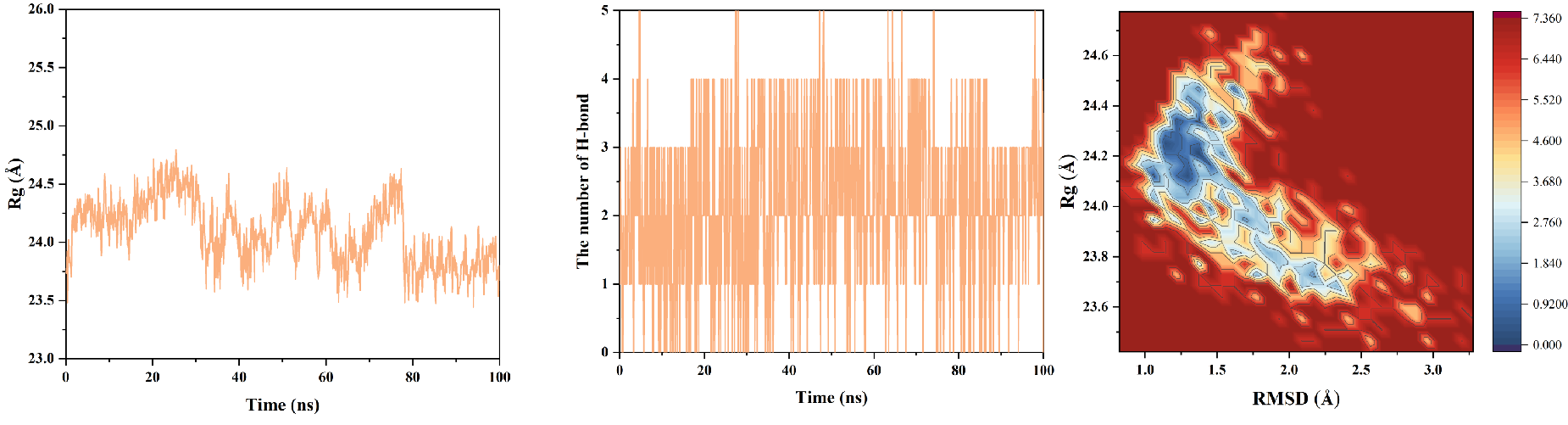


*Fig. S6. Molecular dynamics simulation of the rhein–VCAM1 complex: radius of gyration (Rg), hydrogen bond number curve, and 2D free energy landscape.*


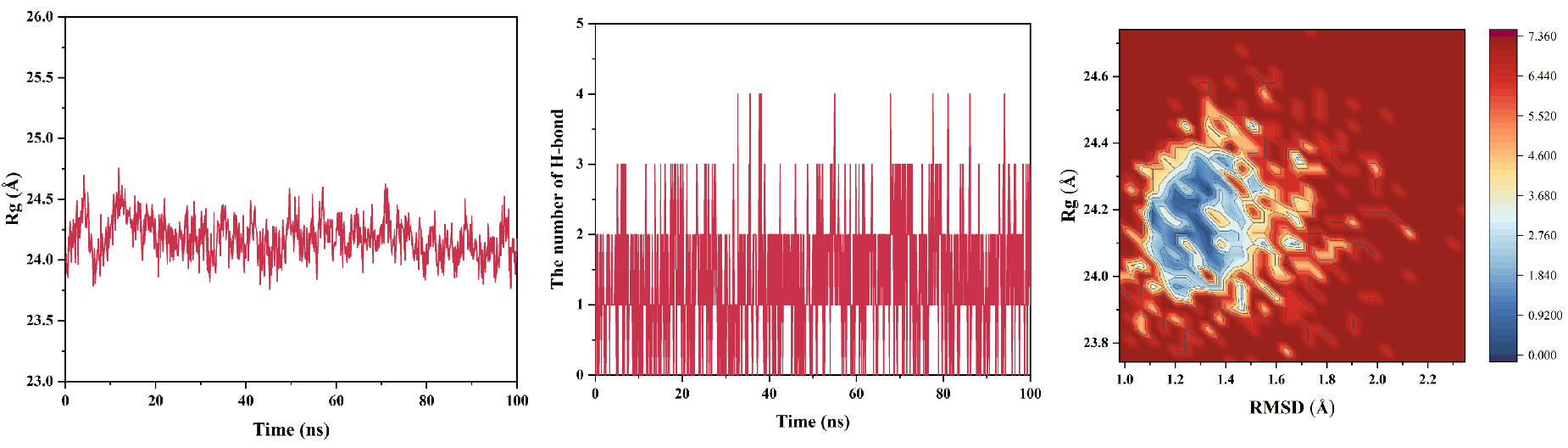


*Fig. S7. Molecular dynamics simulation of the ginsenoside Rg1–VCAM1 complex: radius of gyration (Rg), hydrogen bond number curve, and 2D free energy landscapes.*


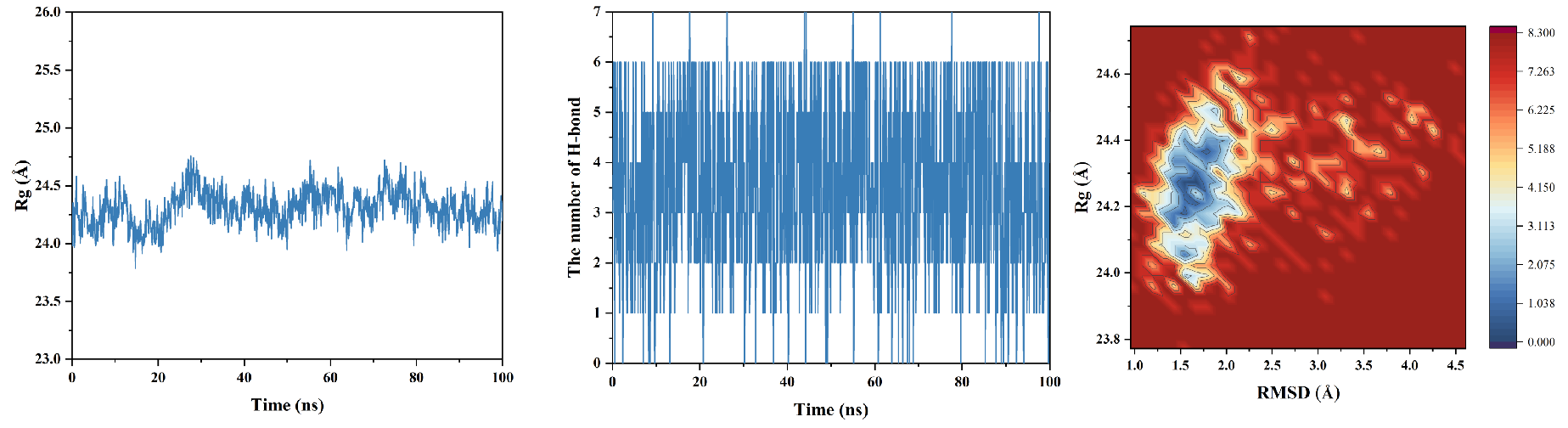


*Fig. S8. Molecular dynamics simulation of the Zingibroside R1–VCAM1 complex: radius of gyration (Rg), hydrogen bond number curve, and 2D free energy landscapes.*
